# Tuning the Cross-Linking Density and Cross-Linker in Core Cross-Linked Polymeric Micelles and Its Effects on the Particle Stability in Human Blood Plasma and Mice

**DOI:** 10.1021/acs.biomac.3c00308

**Published:** 2023-07-14

**Authors:** Tobias A. Bauer, Irina Alberg, Lydia A. Zengerling, Pol Besenius, Kaloian Koynov, Bram Slütter, Rudolf Zentel, Ivo Que, Heyang Zhang, Matthias Barz

**Affiliations:** † Leiden Academic Centre for Drug Research (LACDR), Leiden University, Einsteinweg 55, 2333 CC Leiden, The Netherlands; ‡ Department of Chemistry, Johannes Gutenberg University Mainz, Duesbergweg 10-14, 55128 Mainz, Germany; § Max Planck Institute for Polymer Research, Ackermannweg 10, 55128 Mainz, Germany; ∥ Translational Nanobiomaterials and Imaging Group, Department of Radiology, Leiden University Medical Center, Albinusdreef 2, 2333 ZA Leiden, The Netherlands; # Department of Dermatology, University Medical Center of the Johannes Gutenberg University Mainz, Langenbeckstraße 1, 55131 Mainz, Germany

## Abstract

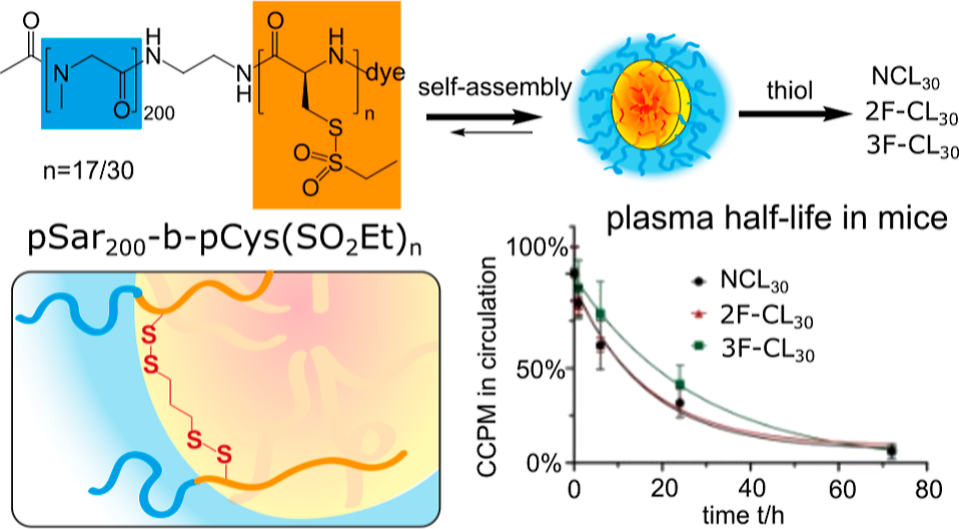

Core cross-linked polymeric micelles (CCPMs) are designed to improve the therapeutic profile of hydrophobic drugs, reduce or completely avoid protein corona formation, and offer prolonged circulation times, a prerequisite for passive or active targeting. In this study, we tuned the CCPM stability by using bifunctional or trifunctional cross-linkers and varying the cross-linkable polymer block length. For CCPMs, amphiphilic thiol-reactive polypept(o)ides of polysarcosine-*block*-poly(*S*-ethylsulfonyl-l-cysteine) [pSar-*b*-pCys(SO_2_Et)] were employed. While the pCys(SO_2_Et) chain lengths varied from *X*_*n*_ = 17 to 30, bivalent (derivatives of dihydrolipoic acid) and trivalent (sarcosine/cysteine pentapeptide) cross-linkers have been applied. Asymmetrical flow field-flow fraction (AF4) displayed the absence of aggregates in human plasma, yet for non-cross-linked PM and CCPMs cross-linked with dihydrolipoic acid at [pCys(SO_2_Et)]_17_, increasing the cross-linking density or the pCys(SO_2_Et) chain lengths led to stable CCPMs. Interestingly, circulation time and biodistribution in mice of non-cross-linked and bivalently cross-linked CCPMs are comparable, while the trivalent peptide cross-linkers enhance the circulation half-life from 11 to 19 h.

## Introduction

Nanomedicine offers the potential to alter the biodistribution of active pharmaceutical ingredients (API) and may provide additional selectivity to potent substances. For hydrophobic drugs, polymeric micelles are the preferred carrier system.^1,2^ Within the core–shell architecture, the drug mainly resides in the inner hydrophobic core, and the hydrophilic corona provides solubility and shielding.^2,3^ Following Nanomedicine 2.0 for drug targeting beyond replacing solubilizers, the carrier and cargo need to be stabilized to prevent premature carrier disintegration and drug release immediately after administration.^4–6^ The primary connection between amphiphilic copolymer and self-assembled polymeric micelle thus needs to be disrupted by either noncovalent kinetic trapping (e.g., by π–π interactions, hydrogen bonding) or dynamic covalent bonds, i.e., by cross-linking.^6–9^ Depending on the cargo, polymeric micelles can be cross-linked by individual strategies. For transition-metal complexes such as platin or ruthenium-based APIs, the drug itself can act as a cross-linker, allowing for drug release upon ligand exchange.^10–13^ Furthermore, click chemistry, amide bond formation, and free radical cross-linking are frequently employed to provide stability to polymeric micelles and allow, among others, for the conjugation of taxane and anthracycline (pro-)drugs.^6,14–16^ Despite the early developed Genexol-PM and Nanoxel, non-cross-linked polymeric micelles (e.g., NK105) could not further demonstrate their superiority in clinical testing.^17,18^ As a result, core cross-linked polymeric micelles (CCPMs) are considered the second generation of polymeric micelles and have evolved to the advanced stages of clinical testing. Currently, CPC634 containing conjugated docetaxel is examined for the treatment of ovarian cancer in clinical phase II, and NC-6004 comprising cisplatin is assessed in phase III for pancreatic cancer therapy.^18,19^

Due to the inherent potential for stable cross-linking yet reversible drug release after cellular uptake, disulfide bonds have attracted significant interest.^6,20^ While disulfide cross-linked micelles can be readily formed from thiol-containing copolymers by oxidation with oxygen in a rather unspecific manner, the reactive *S*-alkylsulfonyl protecting group introduced by Schäfer et al. offers rapid chemoselective disulfide bond formation.^21–24^ When applied to cysteine or homocysteine, the reactive group tolerates nucleophilic amine-initiated *N*-carboxyanhydride (NCA) polymerization and grants access to thiol-reactive polypeptides.^25–27^ The combination of polypeptides with polysarcosine (pSar) in so-called polypept(o)ides is a straightforward approach leading to copolymers entirely based on endogenous amino acids.^28,29^ Polysarcosine, poly(*N*-methyl glycine), is an exclusive hydrogen bond acceptor characterized by a random coil structure in aqueous solution matching all requirements stated by the Whitesides rules.^30,31^ The hydrophilic pSar is thus considered a most promising alternative to poly(ethylene glycol) (PEG) for biomedical applications, showing an improved safety profile such as a reduced induction of cytokine release.^32–36^

Beyond the intended chemical design of a nanoparticle, protein corona formation has been reported to determine the fate of many nanocarriers upon administration into the bloodstream.^37–40^ Unambiguous signs of the protein corona were detected for nanoparticles with sharp and hydrophobic surfaces (e.g., polystyrene and silica nanoparticles) affecting the biological profile.^38,41–43^ For stable nanoparticles with a smoothly decreasing radial density profile, such as CCPMs shielded with a dense corona of either PEG, pSar, or poly(*N*-(2-hydroxypropyl)methacrylamide), conversely, the absence of protein corona formation was observed.^39^ Hereby, analysis by asymmetrical flow field-flow fractionation (AF4) was optimized and used to separate CCPM and CCPM/protein complexes after incubation with human blood plasma followed by high-resolution mass spectrometry.^39^ Separation by AF4 relies solely on the diffusion of the analyte in the separation channel, whereby a parabolic flow profile is combined with an orthogonal cross-flow toward a semipermeable membrane.^44,45^ Depending on Brownian motion, smaller structures elute earlier than larger assemblies or aggregates, while interactions with the static phase are minimized.^44,45^

In contrast to stable CCPMs, the interaction of blood plasma proteins with amphiphilic copolymers originating from insufficiently stabilized polymeric micelles can thus be detected by AF4.^16,44,45^ Non-cross-linked micelles are in constant equilibrium between unimers and micelles. Therefore, the situation is quite complex, and the interaction of plasma proteins with amphiphilic polymers leads to defects in the hydrophilic shell. The defect sites are then prone to unspecific interaction, and the released free polymer may assemble into polymer/protein aggregates.^16,43,46^

The outlined strategies for core cross-linking have already demonstrated the potential to improve the carrier stability and control drug release in vivo.^47^ Nevertheless, up to now, little attention has been paid to the influence of the cross-linking process itself; specifically, how do cross-linker functionality, chemical nature, and the length of the cross-linkable block contribute to the overall particle stability and in vivo performance? Here we investigate the effect of the core size and length and the valency of the cross-linker on the stability of CCPMs prepared from thiol-reactive polysarcosine-*block*-poly(*S*-ethylsulfonyl-l-cysteine) polypept(o)ides using AF4 and fluorescence correlation spectroscopy (FCS) in human blood plasma for evaluation. We further correlate these results to the circulation half-life and biodistribution analysis after intravenous administration of CCPMs to C57BL/6 mice with the view toward defining parameters for CCPM stability.

## Experimental Section

### Materials

All chemicals were purchased from Sigma-Aldrich and used as received, unless stated otherwise. Fmoc-l-cysteine(Trt)–OH, Fmoc-sarcosine, and 2-chlorotrityl chloride-resin were obtained from Iris Biotech GmbH, HFIP and trifluoroethanol (TFE) were sourced from Fluorochem, 1-[bis(dimethylamino)methylene]-1*H*-1,2,3-triazolo[4,5-*b*]pyridinium 3-oxide hexafluorophosphate (HATU) was obtained from Carbolution, deuterated solvents were obtained from Deutero GmbH, and (*R*)-lipoic and was bought from TCI Europe. Atto647N *N*-hydroxysuccinimide (NHS) was obtained from Atto Tec GmbH. Tetrahydrofuran (THF) was dried over sodium and freshly distilled before use. *N*,*N*-Diisopropylethylamine (DIPEA) and *N*,*N*-triethylamine (NEt_3_) were distilled over sodium hydroxide and stored at −20 °C until further use. *N*,*N*-Dimethylformamide (DMF) (99.8%, extra dry over molecular sieves) was purchased from Acros Organics and was subjected to three freeze–pump–thaw cycles to remove dimethyl amine. Milli-Q water was obtained from a Milli-Q Reference A+ System and used at a resistivity of 18.2 MΩ·cm^–1^ and a total organic carbon content below 5 ppm.

### Methods

#### Column Chromatography

Qualitative thin-layer chromatography was performed on silica-coated aluminum sheets (60 Å, F_254_) with a fluorescence indicator from Merck. Analyte absorbance was monitored with UV light (λ = 254 nm). Preparative size-exclusion chromatography (SEC) was performed using a Sephadex LH-20 stationary phase with methanol or chloroform/methanol (1:1) as eluent.

#### Gel Permeation Chromatography

For gel permeation chromatography (GPC), a Jasco GPC setup was used, operating at a flow rate of 1.0 mL min^–1^ at 40 °C. An HFIP solution containing 3 g·L^–1^ potassium trifluoroacetate was used as an eluent and toluene as an internal standard. Three PFG columns in series (particle size 7 μm, porosity 100, 300, and 4000 Å) were used for separation (PSS Polymer Standards Service GmbH, Germany), and poly(methyl methacrylate) standards (PSS Polymer Standards Service GmbH, Germany) and pSar standards were used for calibration.^30^ A UV detector (UV-4070, λ = 230 nm) was used for polymer detection, and data were analyzed using PSS WinGPC.

#### Infrared Spectroscopy

Attenuated total reflectance Fourier transform infrared (ATR-FT-IR) spectroscopy was performed on a FT/IR-4600 spectrometer (Jasco Corporation) equipped with a Jasco ATR Pro ONE unit using the Jasco spectra manager 2.15.18 for data evaluation.

#### Nuclear Magnetic Resonance

The NMR spectra were recorded at room temperature on Avance II 400, Avance III 400, Avance I 500, or Avance III 600 spectrometers (Bruker). DOSY NMR spectra were recorded on a Bruker Avance I 500 using a bipolar pulse program (stebpgp1s) with d20 = 0.2 and p30 = 2750 μs for gradient amplitudes from 5 to 95%. Spectra were calibrated using the solvent signals, and the data were analyzed using MestReNova 14.1.2 (Mestrelab Research S.L.).

#### Single-Angle Dynamic Light Scattering

Dynamic light scattering (DLS) measurements were performed on a Zetasizer Ultra (Malvern Panalytical Ltd.) equipped with a He–Ne laser (λ = 632.8 nm). The samples were measured in phosphate buffered saline (PBS) buffer at 25 °C and at a detection angle of 173° using disposable half-micro polystyrene cuvettes (Carl Roth GmbH & Co. KG, Germany). The cumulant size, polydispersity index (PDI), and size distribution histograms (intensity weighted) were derived from the autocorrelation function using automated position seeking and attenuator selection at multiple scans with a fluorescence filter.

#### Polymer Synthesis

The polymers were synthesized by ring-opening NCA polymerization in anhydrous DMF by using flame-dried glassware and Schlenk techniques. Sarcosine-NCA and *S*-ethylsulfonyl-l-cysteine-NCA were synthesized according to previously published protocols.^21,28^

#### Polysarcosine

The pSar macroinitiator was synthesized according to our previous reports.^48,49^ Briefly, sarcosine-NCA (9.18 g; 79.8 mmol; 220 equiv) was added to a flame-dried Schlenk tube and dissolved in degassed absolute DMF (50 mL). Next, a stock solution of *N*-(*tert*-butoxycarbonyl)-1,2-diaminoethane (58.1 mg; 363 μmol; 1.0 equiv) in dry DMF was added to the Schlenk tube (β = 20 g·L^–1^). The reaction was stirred at 10 °C, shielded from light until the monomer peaks had vanished as determined by IR spectroscopy (9 days). Subsequently, the amine end-group was acylated by overnight stirring with perfluorophenyl-4-azidobutanoate (215 mg; 725 μmol; 2.0 equiv) and DIPEA (308 μL; 1.81 mmol; 5.0 equiv). Next, acetic anhydride (346 μL; 3.63 mmol; 10 equiv) and DIPEA (1.23 mL; 7.25 mmol; 20 equiv) were added, and the reaction was stirred for an additional day at ambient temperature. Next, the polymer was isolated by precipitation in diethyl ether (500 mL) and subsequent centrifugation (4500 rpm; 3 min; 4 °C), whereby the polymer pellet collected and was dried in vacuo. In the following, the Boc-group was cleaved. The crude polymer was transferred to a single-neck round-bottom flask and dissolved in water (25 mL). The solution was cooled to 0 °C, and trifluoroacetic acid (25 mL) was added in one portion. The reaction mixture was stirred at 0 °C for 4 h, after which the solution was transferred into 3 dialysis bags (MWCO, 3.5 kDa) and dialyzed against water (2 medium changes), sodium hydrogen carbonate solution (8 medium changes), and water (8 medium changes). The dialyzed solution was filtered and lyophilized to obtain polysarcosine (**P1**) as a colorless solid (4.04 g, 71%). Complete deprotection was verified by ^1^H NMR (in the absence of the Boc-group signal at 1.37 ppm). The degree of polymerization was determined by HFIP GPC relative to pSar standards (DP = 200). ^1^H NMR (500 MHz, DMSO-*d*_6_): δ (ppm) 4.49–3.80 (m, 2nH, –CH_2_), 3.03–2.66 (m, 3nH, –CH_3_).

#### Polysarcosine-*block*-poly(*S*-ethylsulfonyl-l-cysteine)

The block copolypept(o)ides were synthesized according to our previous reports.^48,49^ The pSar macroinitiator (**P1**) (523 mg; 36.6 μmol; 1.0 equiv) was transferred to a flame-dried Schlenk flask, and the solid was dried by azeotropic distillation with toluene (2×). Subsequently, pSar was dissolved in freshly degassed dry DMF (5.75 mL) and cooled to −10 °C. Then, *S*-ethylsulfonyl-l-cysteine NCA (438 mg; 1.83 mmol; 50 equiv) was added from a stock solution in dry DMF (β = 200 g·L^–1^). The reaction was performed at an overall NCA concentration of β = 55 g·L^–1^. The progress of the reaction was followed by IR spectroscopy, and the reaction was halted after 20 h when a conversion of 38% was detected (correlating to DP 19). The polymer was isolated from solution by precipitation in THF, the obtained suspension was centrifuged (4500 rpm; 5 min; 4 °C), and the supernatant was decanted. The procedure was repeated once with THF, followed by diethyl ether two times. The polymer was dried in vacuo, yielding pSar_200_-*b*-pCys(SO_2_Et)_17_ (**P2**) as a colorless solid (600 mg, 68%). For **P3**, pSar_200_-*b*-pCys(SO_2_Et)_30_, the polymerization was done at an overall NCA concentration of β = 100 g·L^–1^, and the reaction was stopped at 60% conversion. For dye labeling, the copolymer (**P2**, 142 mg; 8.1 μmol; 1.0 equiv) was dissolved in DMSO, Atto647N-*N*-hydroxysuccinimide (2.59 mg; 3.08 μmol; 0.3 equiv) was added, and the solution was stirred at room temperature for 24 h. Subsequently, unconjugated dye was removed by repetitive precipitation in THF (4500 rpm, 3 min, 4 °C), and dye removal was verified by HFIP-GPC. ^1^H NMR (500 MHz, DMSO-*d*_6_): δ (ppm) 8.77 (br s, 1mH, CON*H*), 4.72 (m, 1mH, α–C*H*_(__l__-Cys)_), 4.50–3.79 (m, 2nH, –C*H*_2(Sar)_), 3.54 (m, 4mH, –S–C*H*_2_, –SO_2_–C*H*_2_), 3.06–2.61 (m, 3nH, –CH_3(Sar)_), 1.29 (t, *J* = 7.3 Hz, 3mH, –CH_3(__l__-Cys)_).

#### Cross-Linker Synthesis

The bifunctional cross-linker (*R*)-lipoic acid hydrazide (**3**) was synthesized in a two-step procedure following our previous report (Scheme S1).^49^

##### (*R*)-Methyl Lipoate

(*R*)-Methyl lipoate (**2**) was prepared according to our previous report, and the synthesis was adapted and modified from Hassan and Maltman.^49,50^ (*R*)-Lipoic acid (**1**) (4.00 g; 19.4 mmol; 1.0 equiv) was dissolved in dry methanol (12 mL), and sulfuric acid (10.3 μL; 194 μmol; 0.01 equiv) was added. The reaction mixture was stirred at room temperature for 18 h and protected from light. A yellow solid precipitated after 30 min. The yellow suspension was concentrated in vacuo, and the crude product was dissolved in dichloromethane. The organic phase was washed with NaHCO_3_ solution (3×) and brine (3×), dried over MgSO_4_, filtered, and concentrated in vacuo. (*R*)-Methyl lipoate (**2**) was obtained as a yellow oil (3.84 g; 17.4 mmol; 90%) and used without further purification. ^1^H NMR (400 MHz, DMSO-*d*_6_): δ (ppm) 3.62 (m, 1H, –S–C*H*), 3.58 (s, 3H, -OC*H*_3_), 3.15 (m, 2H, –S–C*H*_2_), 2.40 (m, 1H, –*S*–CH_2_–C*H*_2_), 2.33 (t, *J* = 7.5 Hz, 2H, α–C*H*_2_), 1.87 (m, 1H, –*S*–CH_2_–C*H*_2_), 1.73–1.61 (m, 4H, β–C*H*_2_, δ–C*H*_2_), 1.39 (m, 2H, γ–C*H*_2_).

##### (*R*)-Lipoic Acid Hydrazide

(*R*)-Lipoic acid hydrazide (**3**) was synthesized following our previous report, and the synthesis was adapted and modified from Koufaki et al.^49,51^ (*R*)-Methyl lipoate (**2**) (2.00 g; 9.04 mmol; 1.0 equiv) was dissolved in methanol (10 mL), and hydrazine hydrate (1.33 mL; 27.1 mmol; 3.0 equiv) was added to the yellow solution. The reaction mixture was stirred at room temperature for 96 h in the absence of light. The solution was concentrated in vacuo and dissolved in chloroform. The organic layer was washed with brine (3×), dried with MgSO_4_, filtered, and concentrated in vacuo. (*R*)-Lipoic acid hydrazide (**3**) was obtained as a yellow oil (1.50 g; 6.78 mmol; 75%) and used without further purification. ^1^H NMR (400 MHz, DMSO-*d*_6_): δ (ppm) 8.91 (br s, 1H, –CON*H*), 4.14 (br s, 2H, –NH–N*H*_2_), 3.60 (m, 1H, –S–C*H*), 3.11 (m, 2H, –SC*H*_2_), 2.40 (m, 1H, –*S*–CH_2_C*H*_2_), 2.00 (t, *J* = 7.3 Hz, 2H, α–C*H*_2_), 1.87 (m, 1H, –*S*–CH_2_C*H*_2_), 1.60–1.43 (m, 4H, –β–C*H*_2_, δ–C*H*_2_), 1.40–1.27 (m, 2H, γ–C*H*_2_).

#### Solid Phase Peptide Synthesis

##### Resin Loading

Fmoc-l-cysteine(Trt)–OH (2.0 equiv relative to the resin loading capacity) was dissolved in DCM (10 mL·g^–1^ resin), and a small amount of DMF was added to aid solvation. The vessel (Merryfield Apparatus) was charged with 2-chlorotrityl chloride resin, and the dissolved Fmoc-l-cysteine(Trt)–OH and DIPEA (each 2.0 equiv relative to resin loading capacity) were added. The mixture was shaken for 5 min before additional DIPEA (3.0 equiv) was added and, subsequently, mechanically agitated for 1 h at room temperature. Subsequently, MeOH (1 mL·g^–1^ resin) was added, and the reaction mixture was shaken for 15 min. Next, the solution was drained, and the resin was washed with 10 mL of DCM (3×), DMF (3×), DCM (3×), and MeOH (3×). The loaded resin was dried under a high vacuum overnight.

##### Peptide Synthesis

The following steps were performed on a CS136XT peptide synthesizer (CSBio Ltd.). The loaded resin was placed in a reaction vessel and incubated with DCM to induce swelling. To cleave the Fmoc-group, the DCM was removed, a piperidine solution (20% in DMF) was added, and the vessel was shaken for 20 min. The reaction mixture was drained, and the beads were rinsed multiple times with DMF (4×) followed by DCM (2×). In the next step, a solution of a Fmoc-protected amino acid (4.0 equiv relative to the resin loading capacity) in DMF, HATU (4.0 equiv), and DIPEA (6.0 equiv) was added to the reaction vessel. The reaction mixture was shaken for 4 h. After the reaction had completed, the resin was rinsed with DMF (2×) and DCM (1×), followed by the deprotection step. The Fmoc-deprotection step and the coupling reactions were repeated with respect to the targeted amino acid sequence (NH-Cys-Sar-Cys-Sar-Cys).

##### Cleavage of the Peptide from the Resin

The resin-bound peptide was transferred from the reaction vessel of the peptide synthesizer to a Merryfield Apparatus. A mixture of trifluoroethanol and DCM (2:8; 30 mL) was added, and the beads were shaken for 1 h. The solution was drained, the beads were rinsed with DCM, and the procedure was repeated two times. The peptide containing solutions were combined and concentrated in vacuo. The peptide was precipitated into cold cyclohexane/diethyl ether (2:1), centrifuged, and lyophilized.

##### *N*-*tert*-Butyloxycarbonyl-succinic Acid Monohydrazide (Boc-hydrazine)

Succinic anhydride (**6**) (3.3 g; 33.0 mmol; 1.0 equiv) and Boc-hydrazine (**5**) (4.36 g; 33.0 mmol; 1.0 equiv) were placed in a flask and suspended in H_2_O (60 mL). After 30 min, the solution turned clear. The solution was lyophilized, and *N*-*tert*-butyloxycarbonyl-succinic acid monohydrazide (**7**) (7.63 g; 33.0 mmol; quant) was obtained as a colorless solid. The product was used without any further purification. ^1^H NMR (400 MHz, DMSO-*d*_6_): δ 11.85 (br s, 1H, –COO*H*), 9.53 (s, 1H, –N*H*COCH_2_), 8.68 (s, 1H, –OCON*H*), 2.44–2.37 (m, 2H, –C*H*_2_), 2.33–2.29 (m, 2H, –C*H*_2_), 1.38 (s, 9H, –C*H*_3_). ^13^C NMR (101 MHz, DMSO-*d*_6_): δ (ppm) = 173.6 (–*C*OOH), 170.8 (–NH*C*OCH_2_), 155.3 (–O*C*ONH), 83.6 (–*C*(CH_3_)_3_), 28.7 (–*C*H_2_), 28.1 (–*C*H_3_). ESI-HRMS (MeOH) (*m*/*z*): calcd for [C_9_H_16_N_2_O_5_ + H]^+^, 233.1132; found, 233.1136, calcd for [C_9_H_16_N_2_O_5_ + Na]^+^, 255.0951; found, 255.0958.

##### Boc-hydrazine-Cys(Trt)-Sar-Cys(Trt)-Sar-Cys(Trt)–OH

The peptide was synthesized according to the general procedures on SPPS with 1.72 g (1.6 mmol·g^–1^; 2.75 mmol) resin. The crude product was purified through FC on silica gel (DCM/EtOAc = 1:1 to DCM/EtOAc/MeOH = 5:3:2). After the mixture was freeze-dried, the product (**8**) was obtained as a colorless solid. ^1^H NMR (400 MHz, DMSO-*d*_6_): δ (ppm) 12.81 (s, 1H, –COO*H*), 9.52 (s, 1H, –N*H*COCH_2_), 8.68 (s, 1H, –OCON*H*), 8.60–8.55 (m, 1H, α–CHN*H*), 8.36–8.20 (m, 2H, α–CHN*H*), 7.38–7.19 (m, 45H, –C*H*_arom._), 4.70–4.56 (m, 2H, α–C*H*), 4.26–3.60 (m, 5H, α–C*H*, α_Sar_–C*H*_2_), 2.72–2.62 (m, 6H, –NC*H*_3_), 2.48–2.28 (m, 10H, –C*H*_2_C*H*_2_, –C*H*_2_S), 1.39 (s, 9H, –(C*H*_3_)_3_). ESI-HRMS (MeOH) (*m*/*z*): calcd for [C_81_H_83_N_7_O_10_S_3_ + Na]^+^, 1432.5256; found, 1432.5254.

##### Boc-hydrazine-Cys(Trt)-Sar-Cys(Trt)-Sar-Cys(Trt)-4-fluorobenzylamine

Compound (**8**) (2.46 g; 1.74 mmol; 1.0 equiv) was dissolved in DMF (50 mL) and 4-fluorobenzylamine (654 mg; 5.22 mmol; 3.0 equiv), PyBOP (1.36 g; 2.61 mmol; 1.5 equiv), HOAt (237 mg; 1.74 mmol; 1.0 equiv), and DIPEA (546 μL; 2.09 mmol; 1.2 equiv) were added. The reaction mixture was stirred overnight at ambient temperature. Further PyBOP (453 mg; 871 μmol; 0.5 equiv) and DIPEA (227 μL; 871 μmol; 0.5 equiv) were added and stirred for another 2 h. The solvent was removed in vacuo, the crude product was purified by preparative SEC (CHCl_3_/MeOH = 1:1), and the product (**9**) was obtained as a white solid (1.91 g; 1.74 mmol; 72%). ^1^H NMR (400 MHz, DMSO-*d*_6_): δ (ppm) 9.52 (s, 1H, –N*H*COCH_2_), 8.67 (s, 1H, –OCON*H*), 8.62–8.46 (m, 2H, αCH–N*H*), 8.35–8.17 (m, 2H, -N*H*CH_2_), 7.33–7.19 (m, 47H, –C*H*_arom-Trityl,_ –CH_2_CC*H*_arom_), 7.09–6.96 (m, 2H, –C*H*_arom_CF), 4.69–4.55 (m, 2H, α–C*H*), 4.39–4.24 (m, 3H, α–C*H*, –NHC*H*_2_), 4.06–3.62 (m, 4H, α_Sar_–C*H*_2_), 2.68–2.60 (m, 6H, -NC*H*_3_), 2.43–2.17 (m, 10H, –C*H*_2_C*H*_2_, –C*H*_2_S), 1.38 (s, 9H, –(C*H*_3_)_3_). ^19^F NMR (376 MHz, DMSO-*d*_6_): δ (ppm) −117.4 (CH_arom_C*F*). ESI-HRMS (MeOH) (*m*/*z*): calcd for [C_88_H_89_FN_8_O_9_S_3_ + H]^+^, 1517.5971; found, 1517.5969; calcd for [C_88_H_89_FN_8_O_9_S_3_ + Na]^+^, 1539.5791; found, 1539.5796.

##### Hydrazine-Cys-Sar-Cys-Sar-Cys-4-fluorobenzylamine

The protecting groups of compound (**9**) (1.01 g; 663 μmol; 1.0 equiv) were removed from the peptide with a mixture of TFA/TIPS/H_2_O/ethanedithiol (94:1:2.5:2.5 = 5 mL). The reaction mixture was stirred for 90 min, and in the meantime, the solution turned yellow. Next, the compound was isolated by precipitation in a cold diethyl ether and pentane mixture (1:1), centrifuged, the pellet dried in high vacuum, and freeze-dried. Not all the cleaved protecting groups could be removed through precipitation; therefore, the peptide was solved in water (10 mL) and extracted with diethyl ether (3 × 15 mL). The aqueous layer was lyophilized, and the protecting-group-free product (**10**) was obtained as a colorless solid (392 mg, 488 μmol, 74%). ^1^H NMR (600 MHz, DMSO-*d*_6_): δ (ppm) 10.63 (s, 1H, –N*H*COCH_2_), 8.66–8.64 (m, 0.5H, α–CHN*H*), 8.58–8.55 (m, 1H, α–CHN*H*), 8.49–8.46 (m, 0.5H, α–CHN*H*), 8.39–8.36 (m, 1H, α–CHN*H*), 8.29–8.19 (m, 1H, α–CHN*H*), 7.30–7.28 (m, 2H, –CH_2_CC*H*_arom_), 7.15–7.11 (m, 2H, –C*H*_arom_CF), 4.90–4.78 (m, 1H, α–C*H*), 4.72–4.54 (m, 1H, α–C*H*), 4.44–4.34 (m, 2H, α–C*H*), 4.30–4.27 (m, 2H, –NHC*H*_2_), 4.24–3.82 (m, 4H, α_Sar_–C*H*_2_), 3.08–3.02 (m, 4H, n), 2.86–2.69 (m, 6H, –NC*H*_3_), 2.62–2.52 (m, 4H, –C*H*_2_C*H*_2_), 2.47–2.24 (m, 6H, –C*H*_2_S). ^19^F NMR (376 MHz, DMSO-*d*_6_): δ (ppm) −74.7 (CF_3_), −117.4 (CH_arom_CF). ESI-HRMS (MeOH) (*m*/*z*): calcd for [C_26_H_40_FN_8_O_7_S_3_ + H]^+^, 691.2161; found, 691.2146; calcd for [C_88_H_89_FN_8_O_9_S_3_ + Na]^+^, 713.198; found, 713.197.

##### Polymeric Micelles

The preparation of CCPMs was based on our previous reports with some modifications.^48,49^ Briefly, Atto 647N labeled pSar_200_-*b*-p(l)Cys(SO_2_Et)_17/30_ was dissolved in DMSO (β = 7.5 g·L^–1^) equipped with 1 M thiourea. After 1 h, 20 vol % 1 mM acetate buffer (pH 4.75) containing 10 mM thiourea was added, and the solution was left to equilibrate at room temperature for 4 h. The solution was placed into a dialysis bag and dialyzed with 1 mM acetate buffer (pH 4.75, 10 mM thiourea), and the solvent was changed four times. The solution was filtered by a syringe filter (PVDF; 450 nm) and concentrated to 7 g·L^–1^ by spin filtration (Amicon Ultra; MWCO, 3 kDa), yielding the polymeric micelles (PMs). In the following step, the PMs were treated with (A) (*R*)-dihydrolipoic acid hydrazide (**4**) for 2F-CL_17/30_, (B) the cys/sar pentapeptide (**10**) for 3F-CL_17/30_, or (C) with methyl 3-mercaptopropionate for NCL_17/30_ at equimolar amounts of thiols per *S*-ethylsulfonyl-l-cysteine.

For cross-linking with bifunctional (*R*)-dihydrolipoic acid hydrazide (4), (*R*)-lipoic acid hydrazide (**3**) was dissolved in ethanol at a concentration of β = 20 g·L^–1^, and 0.5 equiv of tris(2-carboxyethyl)phosphine hydrochloride (TCEP·HCl) (β = 50 g·L^–1^ in water) was added. After a reaction for 18 h, the cross-linker solution (**4**) was added to the PMs. For cross-linking with trifunctional Cys/Sar pentapeptide (**10**), the cross-linker was dissolved in ethanol at a concentration of β = 20 g·L^–1^ and added to the PMs. After 48 h of reaction, the CCPM solutions were dialyzed against DMSO/water mixtures (1/1; MWCO, 6–8 kDa) and water, followed by repetitive spin filtration (Amicon Ultra; MWCO, 100 kDa) to remove residual cross-linker and free polymer, as verified by HFIP-GPC.

For quenching with monofunctional methyl 3-mercaptopropionate, an ethanolic solution of thiol-reagent (β = 20 g·L^–1^) was added to the PMs. After 48 h of reaction, the NCL particles were dialyzed against ethanol/water mixtures (1/2; MWCO, 3 kDa) and water, followed by concentration via spin filtration (Amicon Ultra; MWCO, 3 kDa). The final particle concentrations were determined from the lyophilization of aliquots.

##### AF4-Analysis

A 20-fold stock solution of the used PBS was prepared, containing sodium chloride, potassium chloride, disodium phosphate, and potassium phosphate with a final salt concentration of 151.7 and 0.2 mmol/L sodium azide. The stock solution was filtered (Millipore GHP 0.22 μm) for all tests using the AF4 system. Human blood plasma was obtained from the transfusion center of the Medical Department of Johannes Gutenberg-University Mainz. It was pooled from six healthy donors and stabilized with EDTA.

The nanoparticles (30 g/L) were incubated with EDTA-stabilized, pure, and undiluted plasma (1:1, v/v) at 37 °C for 1 h. At the end of incubation, the samples were diluted with PBS to reach a final concentration of 1.5 g/L, corresponding to a 5 vol % solution of plasma in PBS for a sufficient separation. Samples were measured with AF4 immediately after preparation.

The AF4 measurements were performed using an installation from the ConSenxuS GmbH using a constaMETRICR 3200 main pump, a Spectra Series UV150 detector (Thermos Separation), a Dark V3 LS Detector (ConSenxuS GmbH), a Pharmacia P-3500 injection pump, a LV-F flow controller (HORIBA ATEC), and an In-Line Degasser-AF (Waters). A separation channel with a 190 μm spacer and a regenerated cellulose membrane (Mw cutoff: 10 kDa) suitable for protein separation was used.^52^ The UV absorption at 220 nm was monitored. PBS (151.7 mM) containing 0.2 mM sodium azide was used as the eluent for all measurements. The main flow was kept 1 mL/min higher than the cross-flow for each measurement. The cross-flow is illustrated in the respective AF4 elugrams. Every nanoparticle was measured minimally three times independently via plasma independent incubation experiments.

##### FCS-Analysis

The FCS experiments were performed on a LSM 880 (Carl Zeiss, Jena, Germany) setup, equipped with a 633 nm laser and a Zeiss C-Apochromat 40×/1.2 W water immersion objective.^53^ The fluorescence emission was collected with the same objective and directed to a spectral detection unit (Quasar, Carl Zeiss) after it passed through a confocal pinhole. The fluorescence emission was spectrally separated by a grating element on a 32-channel array of GaAsP detectors operating in single-photon counting mode. A detection range of 642–696 nm was used. Each sample was transferred into a well of polystyrene-chambered cover glass (Nunc Lab-Tek, Thermo Fisher Scientific, Waltham, MA) for 15 measurements (10 s for each measurement) at room temperature. In order to examine the behavior of micelles in plasma, the samples were measured after 1 h of incubation with human blood plasma at 37 °C. The obtained time traces were fitted with the following analytical model function
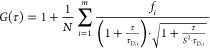
whereby *N* is the average number of the fluorescence species in the detection volume, τ_D,_i is the lateral diffusion time of the *i*-th species, *f*_*i*_ is the fraction of the component *i* (1 ≤ *i* ≤ *m*), and *S* is the structure parameter, *S* = *z*_0_/*r*_0_, where 2*z*_0_ and 2*r*_0_ represent the radius and height of the detection volume, respectively. The diffusion coefficients of species *D*_*i*_ are related to the characteristic diffusion time τ_D,_i and the radial dimension *r*_0_ of *V*_obs_ by *D*_*i*_ = *r*_0_^2^/(4τ_D,_i). The hydrodynamic radius of the respective fluorescent species can be obtained from the Stokes–Einstein equation, . Here, *k*_B_ is the Boltzmann constant; *T* is the temperature, and η is the viscosity of the solvent. As the value of *r*_0_ depends on the optical setup, a calibration was performed using Alexa Fluor 647 (*D* = 330 μm^2^·s^–1^ at 25 °C) as a reference standard with a known diffusion coefficient.

##### Biologic Evaluation

All animal work was performed at the Leiden University animal facility and was approved by the Leiden University Animal Ethics Committee. The animal experiments were performed according to the guidelines from the Dutch government and the Directive 2010/63/EU of the European Parliament on the protection of animals used for scientific purposes under the permit number AVD1060020187085.

Male C57Bl/6 mice, aged 13–17 weeks, were housed in individually ventilated cages under a constant condition with a 12 h light–dark cycle and maintained on a standard mouse diet. Prior to the experiment, mice were weighed and randomly allocated to the different groups using the randomization tool RandoMice, with the weight as a blocking factor for randomization. Mice were acclimatized for 1 week and intravenously injected with 200 μL of Atto647N-labeled CCPMs in PBS (5 μg/μL) or PBS (control) through the tail vein. Blood samples of 50 μL were collected from the tail vein at the defined time points after systemic administration: 10 min, 1 h, 6 h, 24 h, 72 h. EDTA-treated Eppendorf tubes were applied to prevent blood clotting and kept at 4 °C. For fluorescence quantification, blood cells were separated by centrifugation (Mikro 200R, Andreas Hettich GmbH & Co KG) (1000 rpm, 10 min, 4 °C), and 25 μL of the supernatant were transferred into a transparent 96-well plate (Greiner Bio-one, The Netherlands) and diluted with 75 μL of PBS. The particle fluorescence was quantified by a Tecan Spark plate reader (Tecan Group Ltd.) at an emission wavelength of 640 nm and a detection wavelength of 670 nm at a fixed gain of 200 and a bandwidth of 5 nm. The fraction of particles in circulation for each time point was calculated as
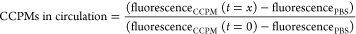


The values obtained at *t* = 10 min were considered as a 100% value.

At 72 h postadministration, mice were euthanized, and after perfusion, the organs were collected and analyzed by ex vivo fluorescence analysis. Lungs, liver, spleen, kidneys, heart, and small intestine were collected and kept in a 6-well plate under PBS stored on ice. The fluorescence per organ was measured on an IVIS Spectrum (PerkinElmer, Massachusetts, USA) using the IS0709N4132 camera (Spectral Instruments TE) (λ_ex_: 605 nm; λ_em_: 680 nm) image acquisition and analysis were performed with Living Image (version 4.7.2; PerkinElmer). For normalization, the total fluorescence intensity was divided by the fluorescent area or the respective organ weight.

## Results and Discussion

**Scheme 1:**
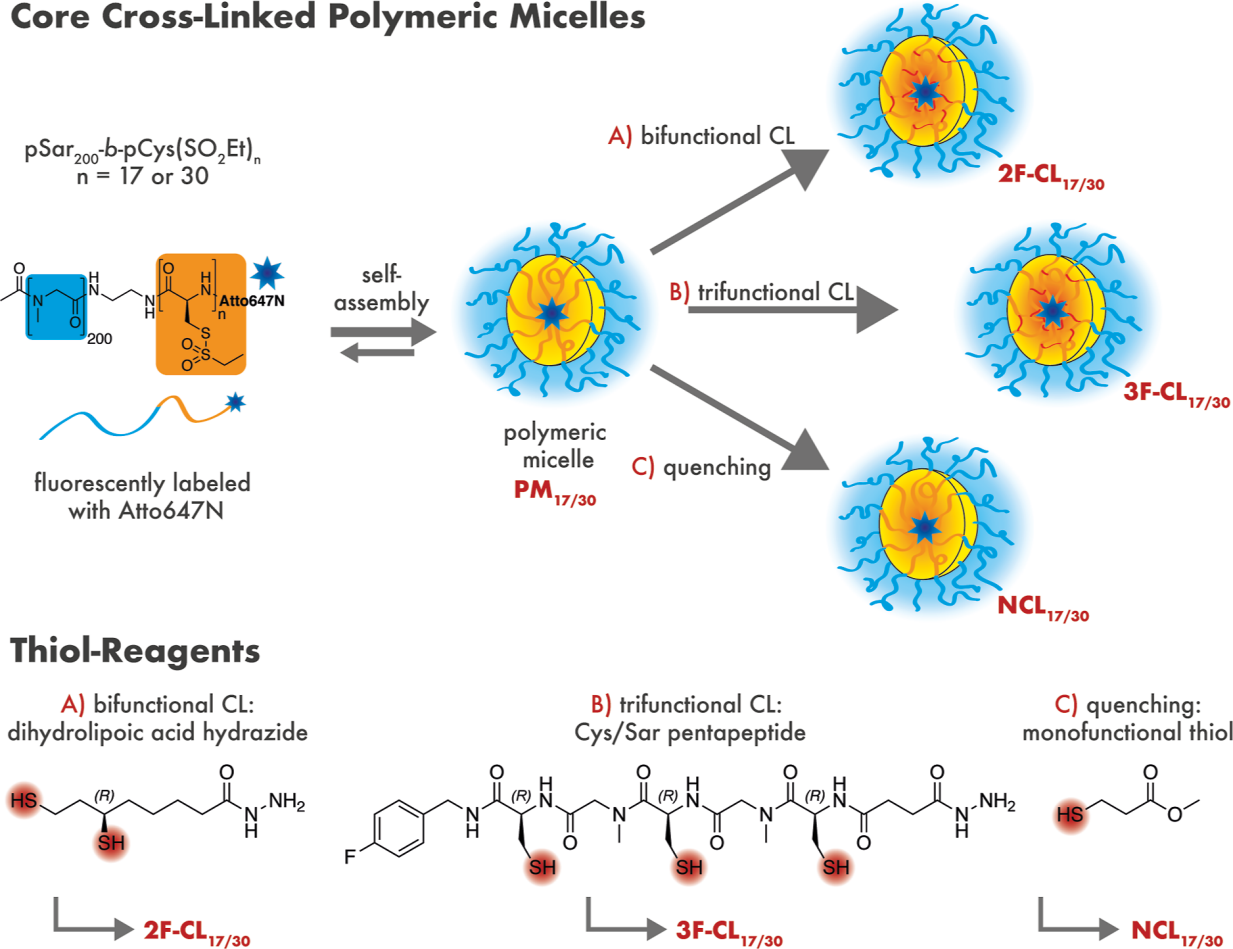
Preparation of CCPMs from pSar-*b*-pCys(SO_2_Et)_*n*_ with *n* = 17 or 30 (PM_17/30_) and Thiol-Reagents of Varying Functionality

Polymeric micelles are frequently applied as carrier systems for hydrophobic drugs, whereby cross-linking has already been demonstrated to improve the circulation time of the nanocarrier.^2,6,47^ In this study, we aimed to investigate whether the cross-linker or the length of the cross-linkable block has a relevant influence on the particle stability in human blood plasma and the circulation time in mice. Therefore, functional cross-linkers were synthesized and CCPMs prepared from polypept(o)ides building up on our previous reports.^48,49,54^ As shown in Scheme 1, polymeric micelles (PMs) were formed by self-assembly of thiol-reactive amphiphilic block copolymers of pSar-*b*-pCys(SO_2_Et). In a second step, the *S*-ethylsulfonyl group was converted by chemoselective disulfide bond formation with the thiol reagents. Thereby, the length of the cross-linkable pCys(SO_2_Et) block was varied from *X*_*n*_ = 17 to 30 (PM_17/30_), whereas the length of the pSar block was kept constant at *X*_*n*_ = 200 to provide sufficient steric shielding. Since cross-linking itself is not sufficient to prevent premature drug release,^55^ hydrazide-modified cross-linkers that grant stimuli-responsive drug conjugation were designed.^56^ Even after the CCPM synthesis, these groups allow for the coupling of ketone-bearing (pro-) drugs such as doxorubicine, epirubicine, or conjugates of taxanes with levulinic acid.^57^ In detail, CCPMs were prepared from bifunctional dihydrolipoic acid hydrazide (2F-CL_17/30_) and the trifunctional cysteine-sarcosine pentapeptide (3F-CL_17/30_). The trifunctional pentapeptide was synthesized by solid-phase peptide synthesis, and the hydrazide linker was introduced via coupling with *N*-*tert*-butyloxycarbonyl-succinic acid monohydrazide in a consecutive step (Scheme S3). The alternating structure of sarcosine and cysteine was selected to provide solubility since pure polycysteine forms insoluble antiparallel β-sheets that complicate the application.^58^ As a control, non-cross-linked micelles were prepared by quenching the *S*-ethylsulfonyl group with monofunctional methyl 3-mercaptopropionate (NCL_17/30_). The reagent was selected based on similar molecular weight and hydrophobicity compared to the *S*-ethylsulfonyl group while considering the odor nuisance and toxicity of small-molecule thiol compounds such as ethanethiol.

**Figure 1:**
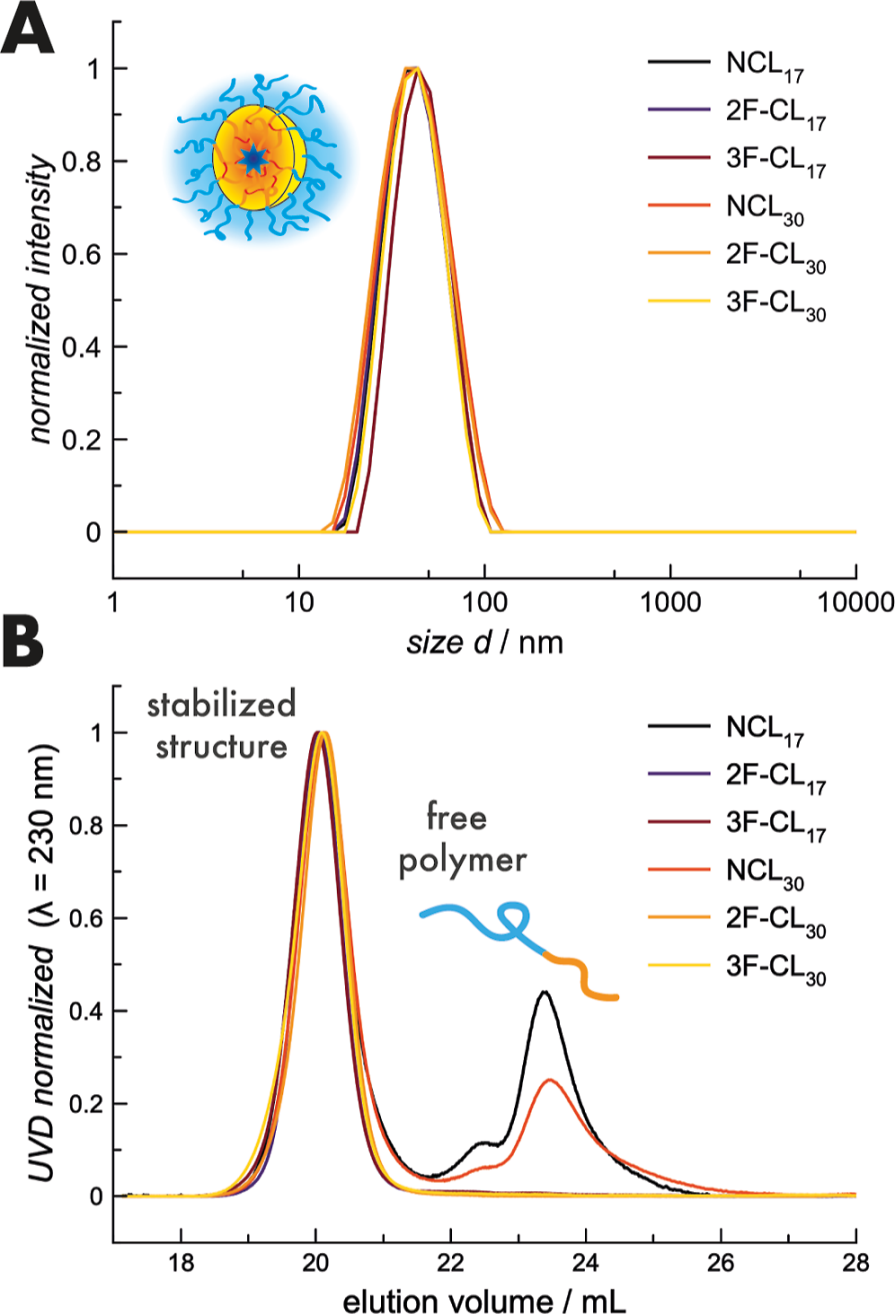
Nanoparticle characterization by DLS (A) and HFIP-GPC (B).

When analyzed by DLS, all particles showed similar hydrodynamic diameters around 40 nm with narrow dispersities of 0.06 to 0.1 (Figure 1). The cross-linking or quenching reaction did thus not affect the overall size distribution, which underlines that neither the chemical nature of cross-linkers nor their valency influences core-size in a detectable manner.^48^ Importantly, for the CCPMs (2F-CL and 3F-CL), only stabilized structures but no unimers could be detected by HFIP-GPC after purification. Vice versa, significant amounts of free polymer could be detected for non-cross-linked micelles. Despite the strong antiparallel β-sheet formation of pCys, which accounts for the stabilized structures correlating with the chain length (free polymer content: NCL_30_ < NCL_17_), a certain degree of cross-linking originating from disulfide-exchange reactions cannot be excluded.^59^ Nevertheless, successful conversion of the *S*-ethylsulfonyl group could be verified by ^1^H NMR, referring to the assigned methoxy group (Figure S2).

**Figure 2:**
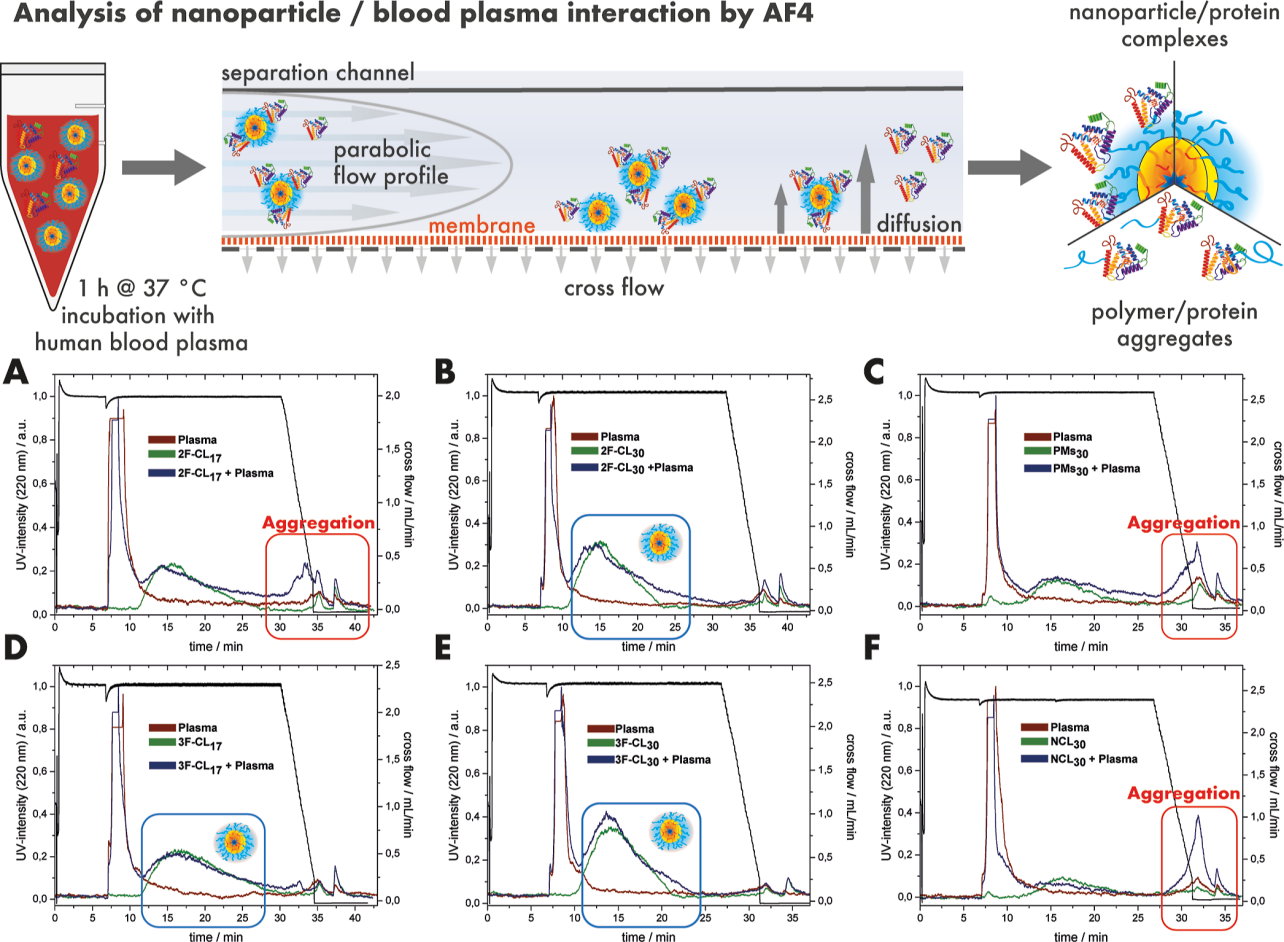
Analysis of CCPMs by AF4 after incubation with human blood plasma. The results of the AF4 analysis as detected by UV absorbance (λ = 220 nm): CCPMs after incubation in PBS (green) or human blood plasma (blue) and plasma controls (red). The following particles have been used: 2F-CL_17_ (A), 2F-CL_30_ (B), PMs_30_ (C), 3F-CL_17_ (D), 3F-CL_30_ (E), and NCL_30_ (F).

In the following, polypept(o)ide-based nanoparticles were analyzed by AF4 and FCS in human blood plasma. The procedure of the AF4 analysis is shown in Figure 2. As illustrated, the samples were incubated in either PBS or human blood plasma for 1 h at 37 °C. Hereby, regenerated cellulose membranes (pore size = 10 kDa) and a cross-flow of up to 2.5 mL/min were applied. Individual cross-flows have been adjusted to individual micelles and therefore differ slightly between individual samples. The isolated micelle–protein complexes or polymer/protein aggregates were then detected based on UV-absorbance and light scattering (LS) intensity. For well-stabilized micelles, like CCPMs, identical elution profiles are expected regardless of the incubation in human plasma.^16,39^

**Figure 3:**
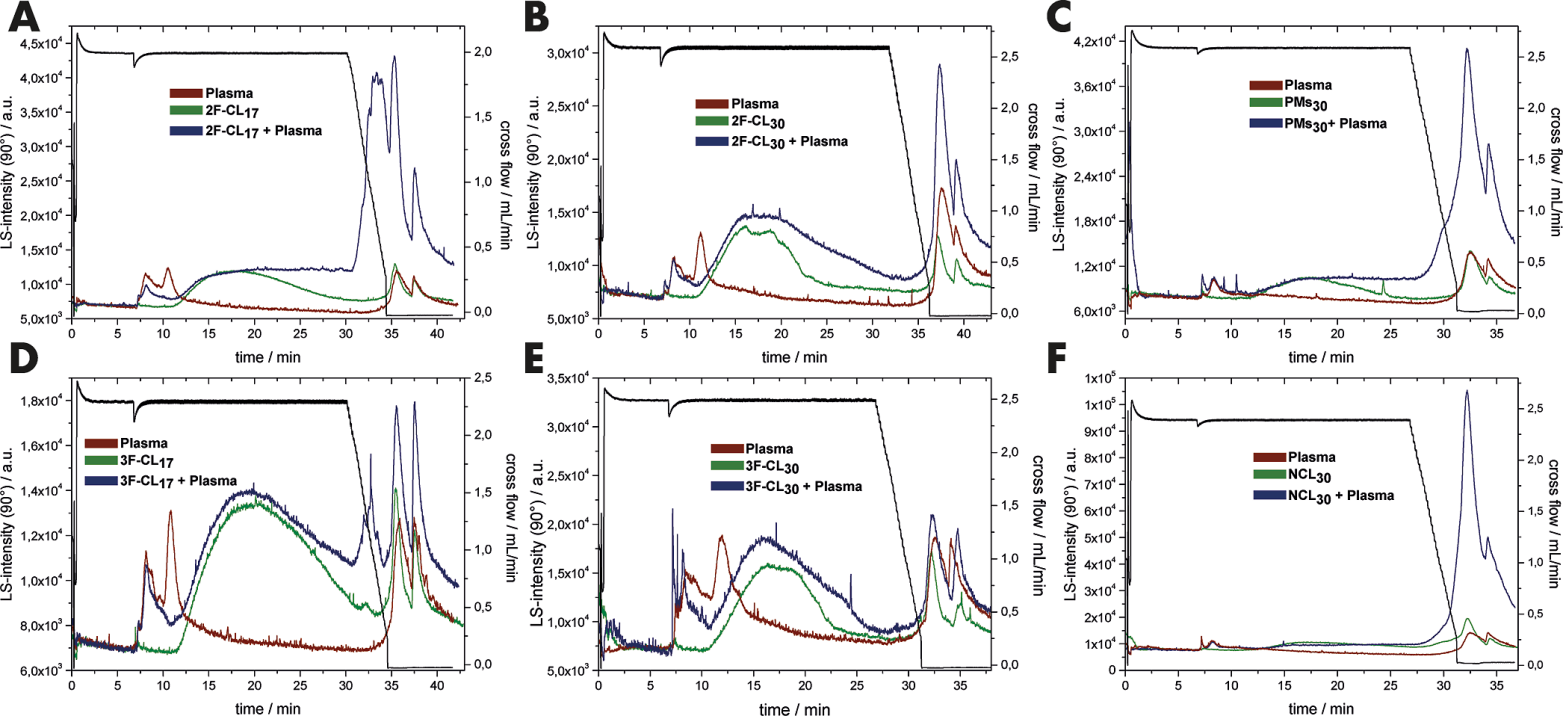
Results of the AF4 analysis as detected by light scattering (scattering angle: 90°): CCPMs after incubation in PBS (green) or human blood plasma (blue), and plasma controls (red). The following particles have been used: 2F-CL_17_ (A), 2F-CL_30_ (B), PMs_30_ (C), 3F-CL_17_ (D), 3F-CL_30_ (E), and NCL_30_ (F).

For all samples incubated in PBS (green color), a distinct particle peak could be identified by the UV-detector at elution times of 10–20 min (Figure 2A–F). After incubation in human blood plasma, however, aggregate formation was detected when the cross-flow was reduced to 0 mL/min and a rinse peak at 30–40 min became visible, as in the case of 2F-CL_17_ (Figure 2A). Since these CCPMs were considered stable previously, displaying the absence of free polymer in HFIP-GPC, the absence of aggregate formation when analyzed by multiangle DLS after incubation in human blood plasma, as well as prolonged circulation time in zebrafish embryos and mice,^54,60^ these findings set the motivation for the detailed study. In fact, when the cross-linking density was enhanced by increasing the number of available net-points, the rinse peak could be reduced significantly following the sequence 2F-CL_17_ < 2F-CL_30_ < 3F-CL_17_ < 3F-CL_30_. Changing the cross-linker functionality from 2 to 3 appears slightly more effective for stabilization than (almost) doubling the cross-linkable polymer block, which reflects the gel-point theory for the polymerization of multifunctional monomers (Carothers equation). In addition, for the CL_30_ species, the particle peak became slightly broader after incubation with blood plasma, which could indicate an increased protein corona formation or particle insatbility.^39^ On the other hand, for PM_30_ and NCL_30_, the particle peak almost entirely vanished after incubation in blood plasma, leading to a strongly elevated rinse peak (Figure 2C, F). Beyond detection by UV absorbance, light scattering is even more sensitive to large structures, providing extra resolution to AF4. As shown in Figure 3, the insufficiently stabilized samples 2F-CL_17_, PM_30_, and NCL_30_ show almost no particle peak but only a large fraction of aggregates after incubation with human blood plasma. Furthermore, the rinse peak practically disappeared for 3F-CL_30_, with intermediate intensities for 2F-CL_30_ > 3F-CL_17_, supporting the findings from the UV-detection.

**Figure 4:**
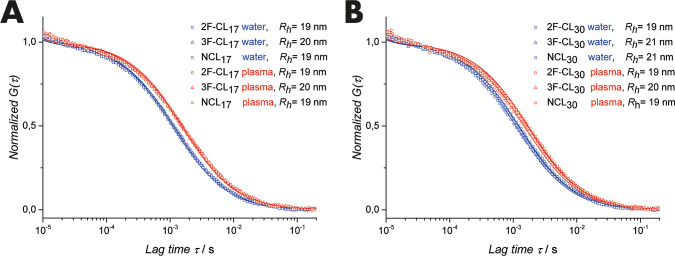
Results of the FCS analysis in water (blue) and plasma (red): the normalized autocorrelation functions *G*(τ) are given for nanoparticles prepared from pSar-*b*-pCys(SO_2_Et)_17_ (A) or pSar-*b*-pCys(SO_2_Et)_30_ (B) and monofunctional, bifunctional, or trifunctional cross-linkers. The hydrodynamic radii were derived via the Stokes–Einstein relation.

Opposing on the trend of the micelle stability revealed by AF4, analysis by FCS in human blood plasma did not show any differences among the samples (Figure 4). In aqueous solution, hydrodynamic radii from 19 to 21 nm were detected for all particles, which is in line with the results from DLS. In addition, no remaining free dye (Atto647N) could be detected for all samples. However, the exact same radii were calculated after incubation with human blood plasma, regardless of cross-linking (2F-CL_17/30_ and 3F-CL_17/30_) or quenching (NCL_17/30_). FCS is a precise method to determine the size of colloids, nanoparticles, or proteins.^61^ Moreover, following the procedure established by Negwer et al., FCS can even be applied directly in human blood.^62^ FCS relies on the diffusion of the fluorescent probe through the small confocal observation volume. The diffusion coefficient can be derived from the autocorrelation function translating to the hydrodynamic radius via the Stokes–Einstein relation. Alterations in the radius or the quality of the fit indicate interaction, aggregation, or protein corona formation with high sensitivity.^62,63^ The unchanged radii of 19 to 21 nm thus indicate stable particles in both conditions.

**Figure 5:**
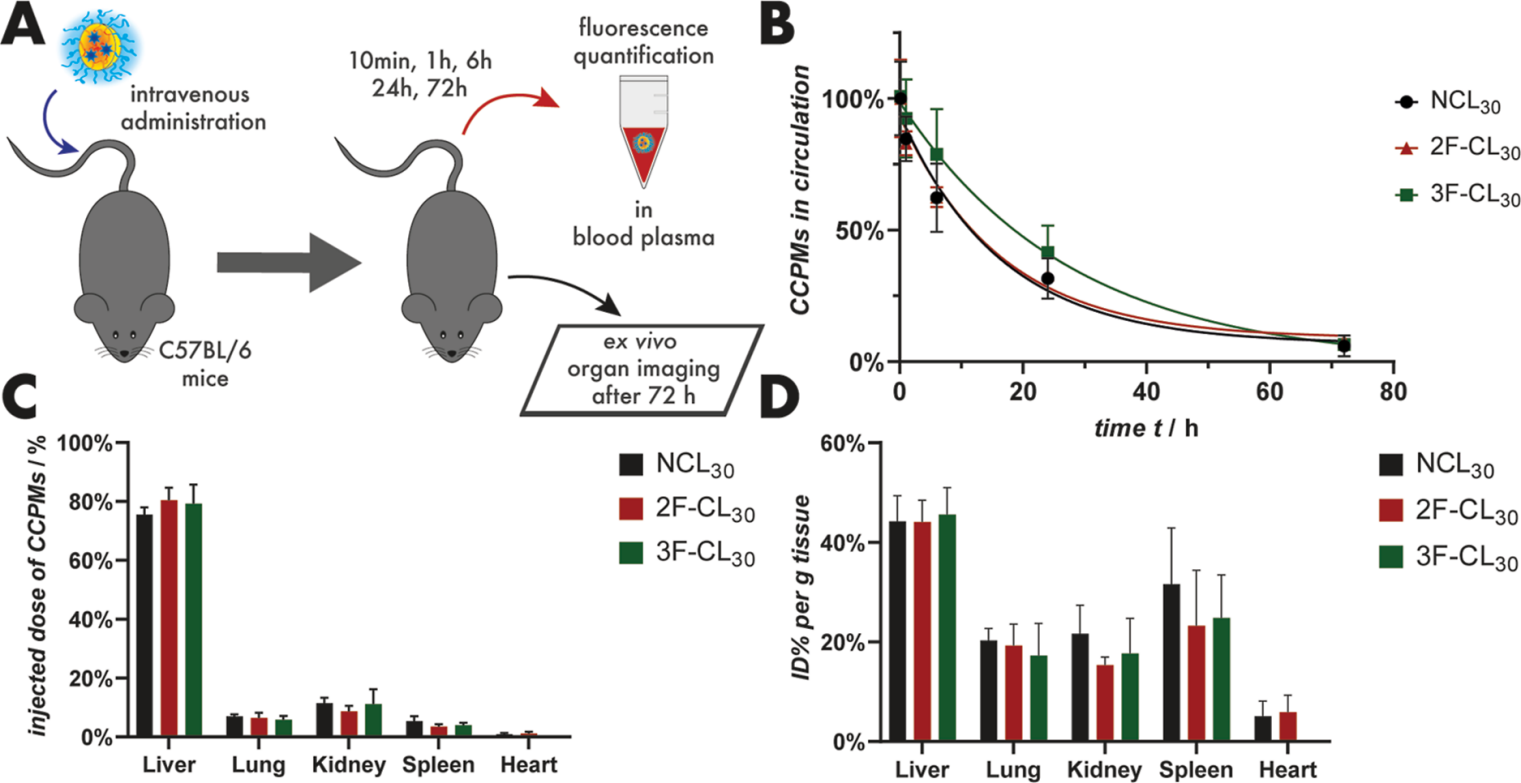
Evaluation of cross-linked and non-cross-linked polymeric micelles in C57BL/6 mice. Schematic illustration of the in vivo experiment (A). Nanoparticle circulation time analysis was calculated from the nanoparticle fluorescence in blood plasma samples. Normalization to 10 min time point, fit model: monoexponential decay (B). Nanoparticle biodistribution in the liver, lungs, kidneys, spleen, and heart after 72 h post-injection based on the total fluorescence intensity in ex vivo organ imaging (C). Nanoparticle biodistribution in the liver, lungs, kidneys, spleen, and heart 72 h post-injection was based on the fluorescence intensity relative to the organ weight in ex vivo organ imaging (D).

To relate the contradictory results of the two screening techniques to the in vivo situation, the cross-linked (2F-CL_30_ and 3F-CL_30_) and non-cross-linked (NCL_30_) nanoparticles were investigated for their circulation time and biodistribution in mice. As displayed in Figure 5A, the micelles were administered to C57BL/6 mice by intravenous injection, and blood samples were taken at the indicated time intervals and analyzed for nanoparticle-associated fluorescence. In addition, the tissue exposure was measured by ex vivo organ imaging 72 h post-injection. The results of the in vivo study are displayed in Figure 5, and all screening data are summarized in Table 1.

**Table 1:** Summary of the Nanoparticle Properties and Blood Half-Life Time

particle	*D*_h_/nm^a^	PDI^a^	*R*_h_/nm^b^	*R*_h_/nm^c^	AF4-score^d^	*t*_1/2_/h^e^	CI (*t*_1/2_)/h^e^
NCL_17_	39	0.06	19	19			
2F-CL_17_	39	0.09	19	19	3		
3F-CL_17_	43	0.08	20	20	2		
NCL_30_	40	0.09	21	19	4	11.4	6.8–18.7
2F-CL_30_	38	0.10	19	19	2	11.3	7.4–17.1
3F-CL_30_	40	0.07	21	20	1	19.1	12.3–34.8

a DLS in PBS.

b FCS in PBS.

c FCS with human plasma.

d AF4 with human plasma, qualitative score: (1): no interaction detectable, (2): slight rinse peak detectable, (3): large rinse peak detectable, (4): large rinse peak detectable, and particle peak vanished.

e Intravenous administration in C57BL/6 mice, fluorescence detection from plasma samples, fit model: monoexponential decay.

For all particle groups, circulation half-lives of 11.3–19.1 h were recorded. Interestingly, no clear trend could be derived among the treatment groups. Within the error of the mean (*N* ≥ 4) and the calculated 95% confidence intervals (CI), no significant increases in fluorescent signal could be detected for any distinct time point nor for the resulting circulation half-life. Nevertheless, compared to NCL_30_ and 2F-CL_30_, 3F-CL_30_ showed a slightly decreased CCPM clearance as higher particle contents in blood could be detected at 6 h (*p* ≤ 0.265) and 24 h (*p* ≤ 0.261) post-administration. For the accumulation of the nanoparticles in the liver, lung, kidney, spleen, and heart, no significant differences could be detected among the different micelles. Relative to the tissue weight, approximately 40 and 30% can be detected in the liver and spleen, respectively. Since the liver is the major organ for nanoparticle clearance, the predominant accumulation contributes to an improved toxicity profile of drugs with high toxicity to the heart (doxorubicine) or the kidneys (cisplatin), while the long circulation time sets the basis for passive tumor accumulation via the EPR effect.^18,64,65^

Taken together, we applied AF4 and FCS to determine the stability of disulfide cross-linked polymeric micelles in human blood plasma with the aim of determining structure–activity relationships predicting the in vivo fate. AF4 analysis revealed a very detailed image of the micelle properties, clearly linking the number of cross-linkable groups to the tendency to form polymer/protein aggregates. Conversely, FCS analysis in human plasma did not indicate any aggregation or undesired interaction of nanoparticles and blood plasma components. In a similar manner, upon intravenous administration, no differences could be detected for the circulation half-life or biodistribution of cross-linked or non-cross-linked particles based on pSar-*b*-pCys(SO_2_Et). Furthermore, the 10 min time point, which was used for normalization, may have been selected too late, since initial drop values are typically observed for structures without additional stabilization.^6,66^ Of note, even for the NCL particles, additional stabilization by antiparallel β-sheets needs to be taken into account, as well as possible cross-linking by disulfide shuffling, as mentioned above.^49,58,59^ Despite the clear improvement of the particle stability as recognized by AF4 upon increasing the cross-linker functionality and the length of the cross-linkable section, it remains challenging to derive the quantitative means. In particular, light scattering is very sensitive to large structures (*I* ∼ *r*^6^), potentially overrunning a small fraction of the sample. On the other hand, both FCS and the in vivo experiment only refer to the particle fluorescence and thus a number-weighted result, in which a small species may simply be overlooked. But there is another important factor to be considered, regenerated cellulase membranes are treated with CS_2_ for their synthesis, leaving traces of this highly reactive component (and possibly some thiols) in the membrane behind. They can, later on, diffuse out of the membrane, interfere with disulfide bonds and thus reduce cross-linking density or modify protein surfaces. In contrast to our former studies on other CCPMs, only disulfide-based CCPMs are affected, in which a partial disulfide bond cleavage can occur. Therefore, these impurities might explain the observed reduced stability of disulfide CCPMs. Since we were unable to get information or conclusive experimental data on the CS_2_ content of the regenerated cellulose membranes applied in this study, we can only recommend considering AF4 analysis of disulfide-based CCPMs with great care whenever regenerated cellulose membranes are used. On the other hand, such membranes have displayed a superior applicability in serum measurements compared to several other membranes.^39^ Therefore, future research needs to address the use of alternative membranes or the detailed characterization and subsequent reduction of CS_2_ content in regenerated cellulose membranes.

Nevertheless, our findings provide valuable insights into nanoparticle stability in human plasma and the detection thereof by combining different analytical methods. Considering the careful development of a medicinal product, all signs of aggregation or interaction of the product with blood plasma components need to be taken very seriously, as they may affect patient compliance or even cause severe toxicity.

## Conclusions

We investigated CCPMs for their stability in human blood plasma by AF4 and FCS and correlated the results to the biodistribution and circulation half-life after intravenous administration in C57BL/6 mice. Based on the thiol-reactive polypept(o)ides of pSar_200_-*b*-pCys(SO_2_Et), the length of the cross-linkable pCys(SO_2_Et) block was chosen 17 or 30. The polymeric micelles were cross-linked by linkers with varied functionality. Bifunctional dihydrolipoic acid hydrazide and a trifunctional Cys/Sar pentapeptide were used to generate core cross-linked particles. Monofunctional methyl 3-mercapto propionate was applied to convert the reactive *S*-ethylsulfonyl group into a disulfide bond without any cross-linking. After incubation in human blood plasma, AF4 analysis revealed a clear connection between the nanoparticle stability and the number of net points or the cross-linker functionality. Clear signs of aggregation could be detected for non-cross-linked structures. Opposing on these results, FCS analysis in human blood plasma did not detect any signs of aggregation or protein corona formation for any sample. Moreover, a similar biodistribution and comparable circulation half-times of 11.3–19.1 h were found for all nanoparticles, indicating no significant differences. The observed variances may be attributed to the sensitivity and detection modes of the analytical techniques or CS_2_ contamination in regenerated cellulose membranes. Nevertheless, the presented combination of analytical techniques demonstrates how the stability of CCPMs can be analyzed and adjusted efficiently.

## Supporting Information Available

The following files are available free of charge. The Supporting Information is available free of charge at https://pubs.acs.org/doi/10.1021/acs.biomac.3c00308.

Cross-linker synthesis and NMR analysis, HFIP GPC analysis of pSar_200_-*b*-pCys_17/30_, NMR spectra of NCL_30_ and PM_30_; ex vivo images of isolated organs from mice; and NMR spectra of pSar_200_-*b*-p(L)Cys(SO2Et)_17/27_ (PDF)
