# Supplementary material for: Tuning the Cross-Linking Density and Cross-Linker in Core Cross-Linked Polymeric Micelles and Its Effects on the Particle Stability in Human Blood Plasma and Mice
Source: Biomacromolecules. 2023 Jul 14;24(8):3545–56. doi: 10.1021/acs.biomac.3c00308 (PMC10428167; doi:10.1021/acs.biomac.3c00308)
Supplement: Supplementary file 1 — bm3c00308_si_001.pdf [file bm3c00308_si_001.pdf]

Supporting Information

Tuning the cross-linking density and cross-linker in core-crosslinked polymeric micelles and its effects on the particle stability in human blood plasma and mice

Tobias A. Bauer, Irina Alberg, Lydia A. Zengerling, Pol Besenius, Kaloian Koynov, Bram Slütter, Rudolf Zentel, Ivo Que, Heyang Zhang, Matthias Barz*

Affiliations

Tobias A. Bauer - *Leiden Academic Centre for Drug Research (LACDR), Leiden University, Einsteinweg 55, 2333 CC Leiden, The Netherlands*; Email: t.a.bauer@lacdr.leidenuniv.nl

Irina Alberg - *Department of Chemistry, Johannes Gutenberg University Mainz, Duesbergweg 10-14, 55128 Mainz, Germany*, Email: ialberg@uni-mainz.de

Lydia A. Zengerling - *Department of Chemistry, Johannes Gutenberg University Mainz, Duesbergweg 10-14, 55128 Mainz, Germany*; Email: zengerling@uni-mainz.de

Pol Besenius - *Department of Chemistry, Johannes Gutenberg University Mainz, Duesbergweg 10-14, 55128 Mainz, Germany*; Email: besenius@uni-mainz.de

Kaloian Koynov - *Max Planck Institute for Polymer Research, Ackermannweg 10, 55128 Mainz, Germany*; Email: koynov@mpip-mainz.mpg.de

Bram Slütter - *Leiden Academic Centre for Drug Research (LACDR), Leiden University, Einsteinweg 55, 2333 CC Leiden, The Netherlands*; Email: b.a.slutter@lacdr.leidenuniv.nl

Rudolf Zentel - *Department of Chemistry, Johannes Gutenberg University Mainz, Duesbergweg 10-14, 55128 Mainz, Germany*; Email: zentel@uni-mainz.de

Ivo Que - *Translational Nanobiomaterials and Imaging Group, Department of Radiology, Leiden University Medical Center, Albinusdreef 2, 2333 ZA, Leiden, the Netherlands*; Email: i.que@lumc.nl

Heyang Zhang - *Leiden Academic Centre for Drug Research (LACDR), Leiden University, Einsteinweg 55, 2333 CC Leiden, The Netherlands*; Email: h.zhang@lacdr.leidenuniv.nl

Matthias Barz - *Leiden Academic Centre for Drug Research (LACDR), Leiden University, Einsteinweg 55, 2333 CC Leiden, The Netherlands; Department of Dermatology, University Medical Center of the Johannes Gutenberg University Mainz, Langenbeckstraße 1, 55131 Mainz, Germany*; Email: m.barz@lacdr.leidenuniv.nl

Cross-Linker Synthesis

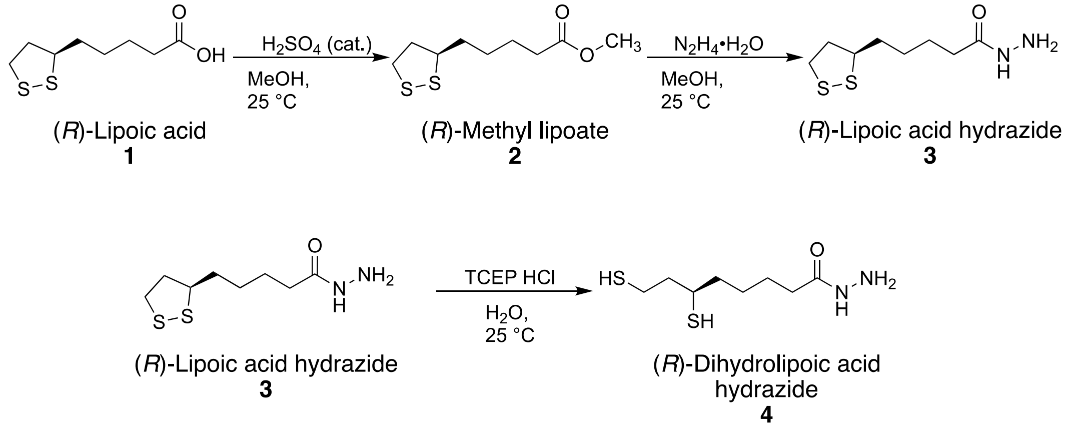

Scheme S1. Synthetic pathway to the bifunctional lipoic acid hydrazide (**4**) cross-linker.

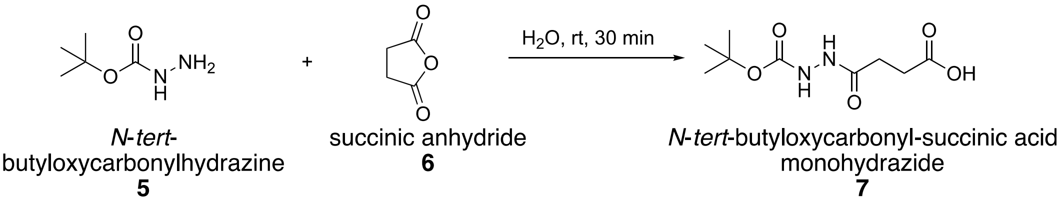

Scheme S2. Synthetic pathway to the N-tert-butyloxycarbonyl-succinic acid monohydrazide (**7**).

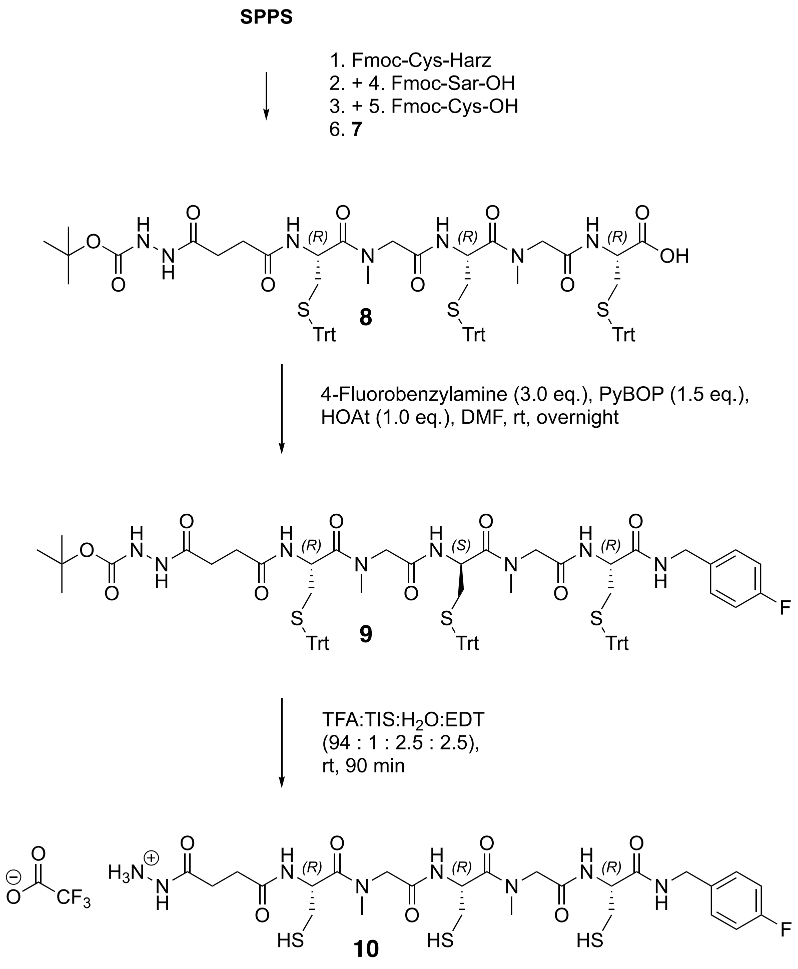

Scheme S3. Synthetic pathway to the trifunctional pentapeptide cross-linker **10** via solid-phase peptide synthesis.

Polymer Synthesis

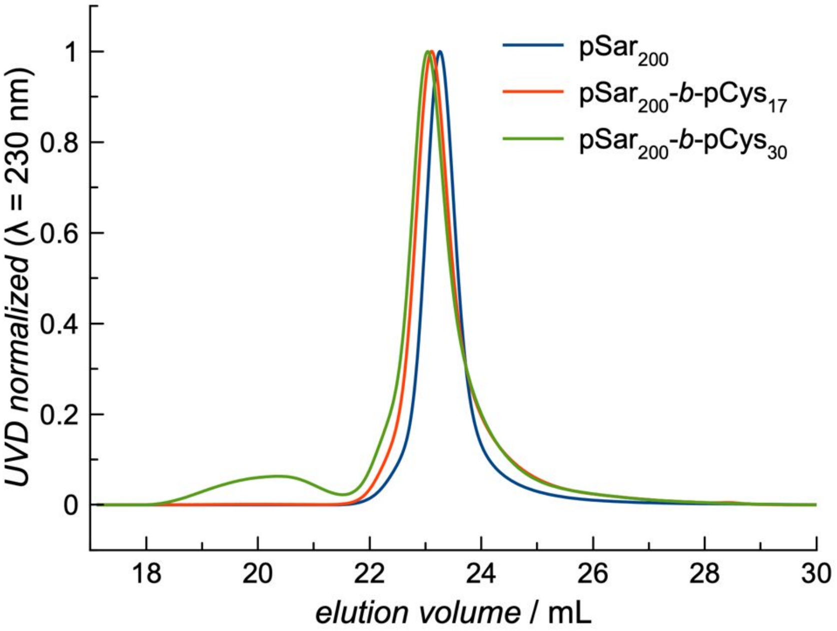

Figure S1. Analytical HFIP GPC of polypept(o)ides. Note that secondary structure formation (anti-parallel β-sheet) is not suppressed in HFIP accounting for the broad PDI of copolymers with increasing chain length of p(L)Cys(SO_2Et).

Particle Synthesis

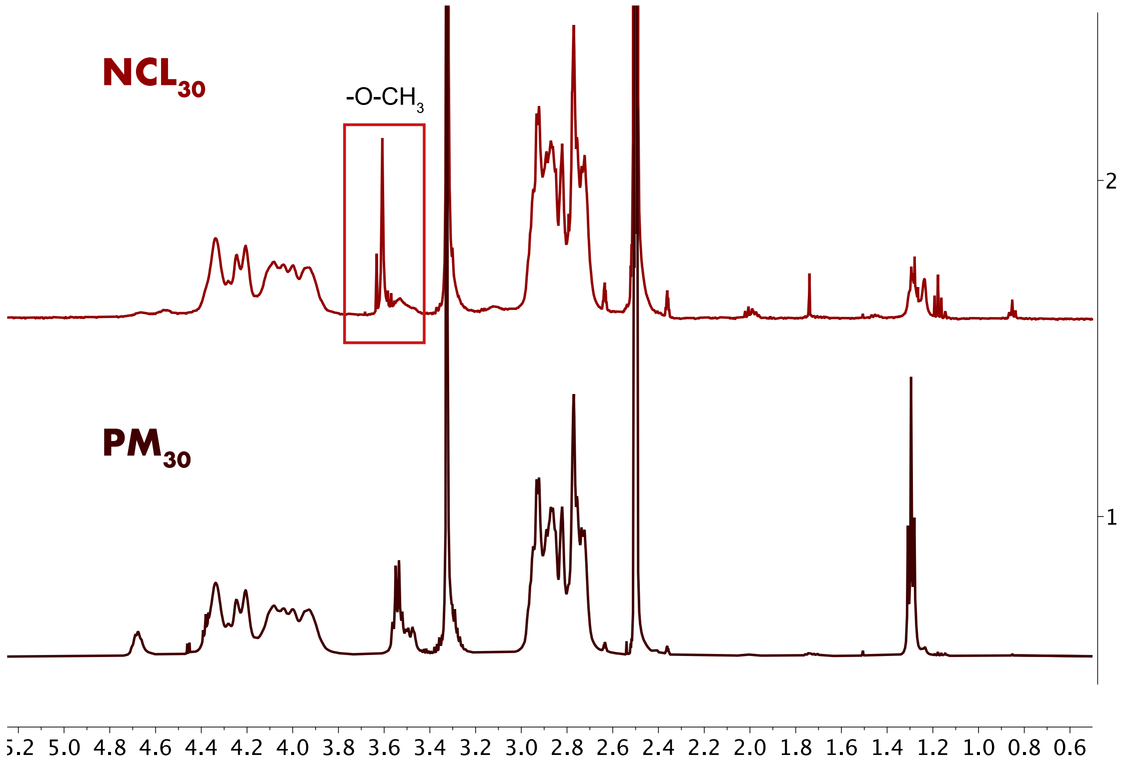

Figure S2. NMR analysis of the polymeric micelles before (PM_{30}) and after quenching of the *S*-ethylsulfonyl group with methyl 3-mercaptopropionate (NCL_{30}). For NCL_{30}, no signals of the *S*-ethylsulfonyl group but only signals of the methoxy group can be detected indicating complete removal of the reactive group.

***Ex vivo* Imaging**

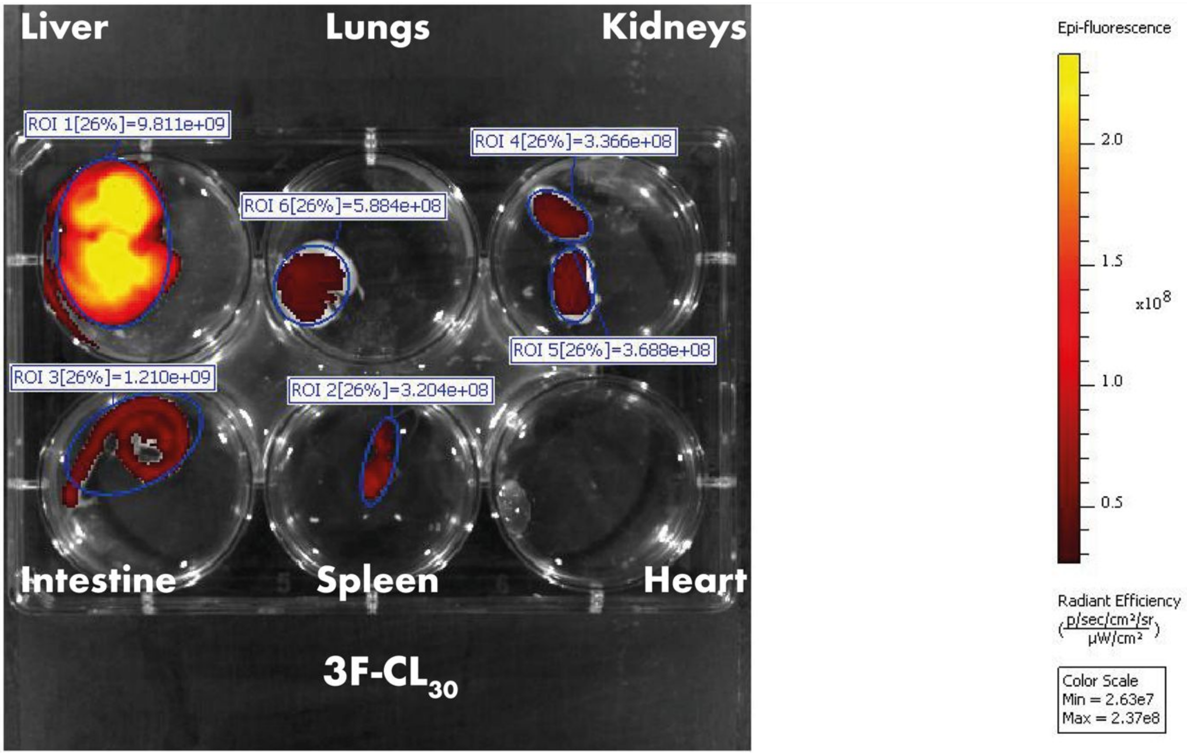

Figure S3. *Ex vivo* organ imaging by IVIS Spectrum (Perkin Elmer, Massachusetts, USA) shown for a representative mouse treated with 3F-CL_{30}. Fluorescence associated to the intestine originates from the diet.

NMR Spectroscopy

Polypept(o)ides

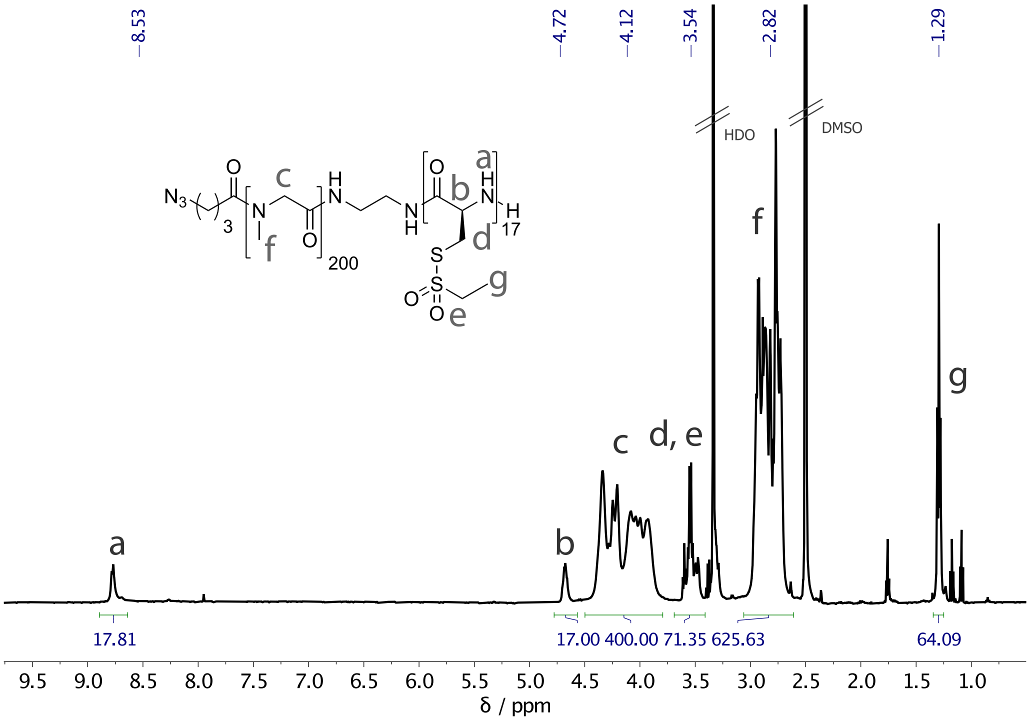

Figure S4. ^{1}H NMR of pSar$_{200}$-*b*-p(L)Cys(SO$_2$Et)$_{17}$ in DMSO-*d*$_6$.

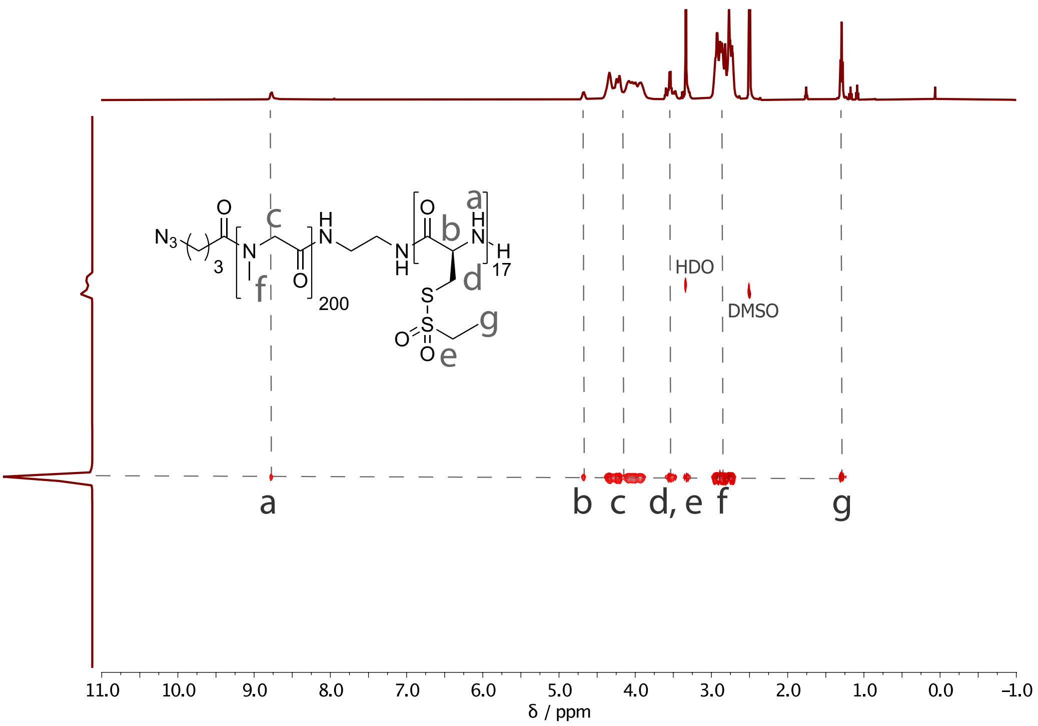

Figure S5. DOSY NMR Analysis of pSar$_{200}$-*b*-p(L)Cys(SO$_2$Et)$_{17}$ in DMSO-d_6.

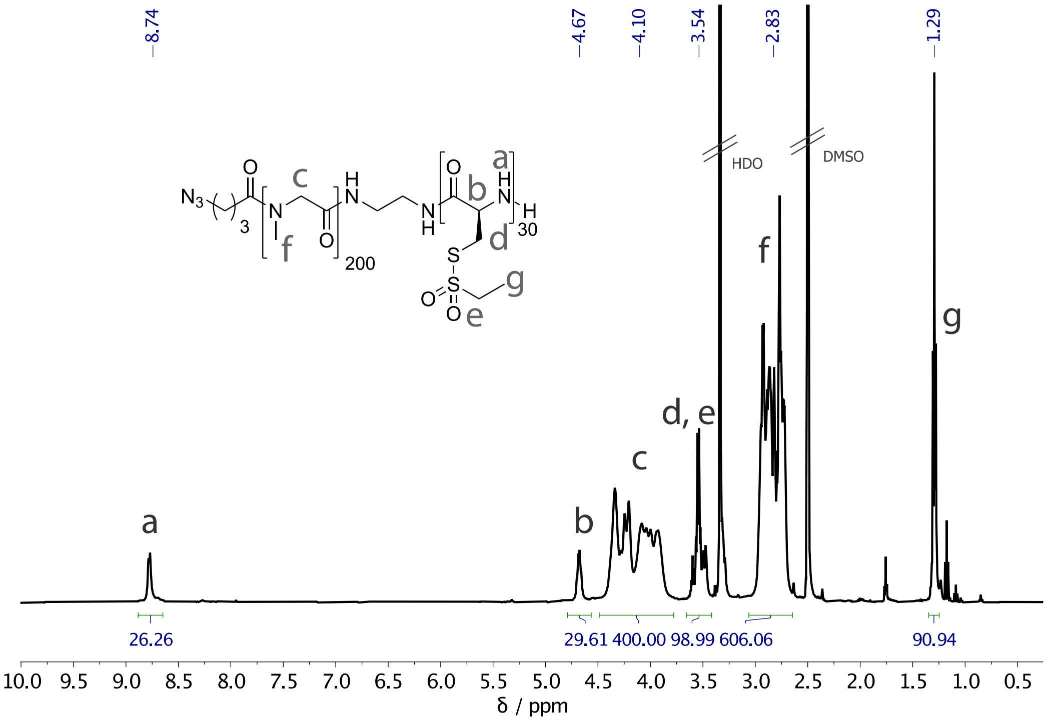

Figure S6. ^{1}H NMR of pSar$_{200}$-*b*-p(L)Cys(SO$_2$Et)$_{27}$ in DMSO-d_6.

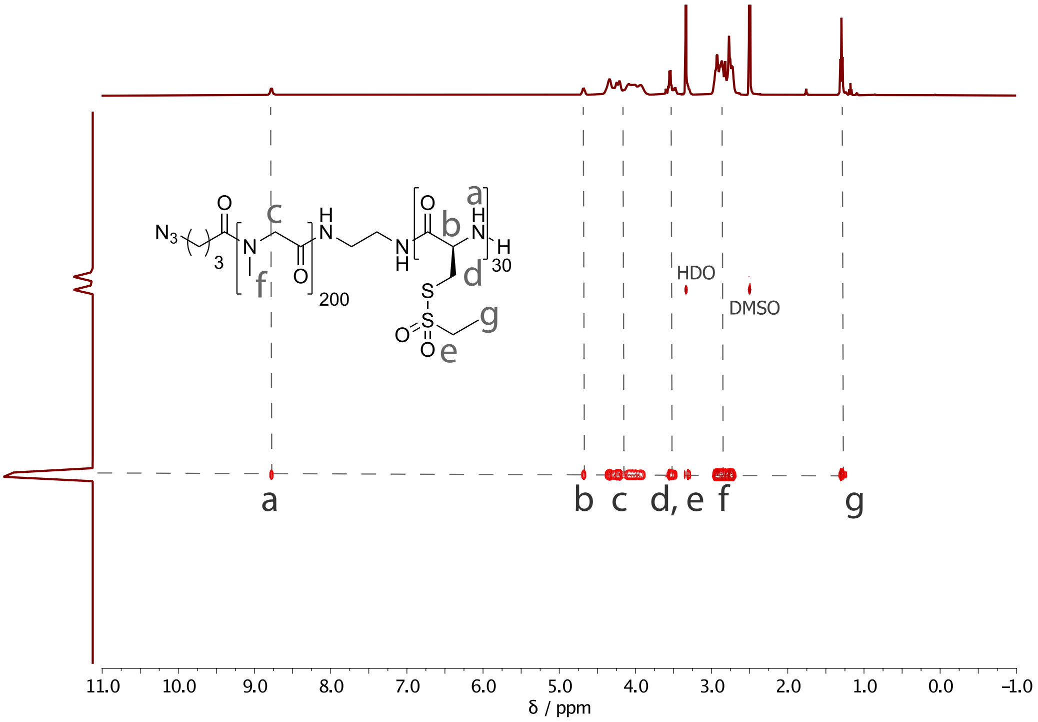

Figure S7. DOSY NMR Analysis of $pSar_{200}$-*b*-p(L)Cys(SO_2Et)$_{27}$ in DMSO-d_6.

Peptide Cross-Linker

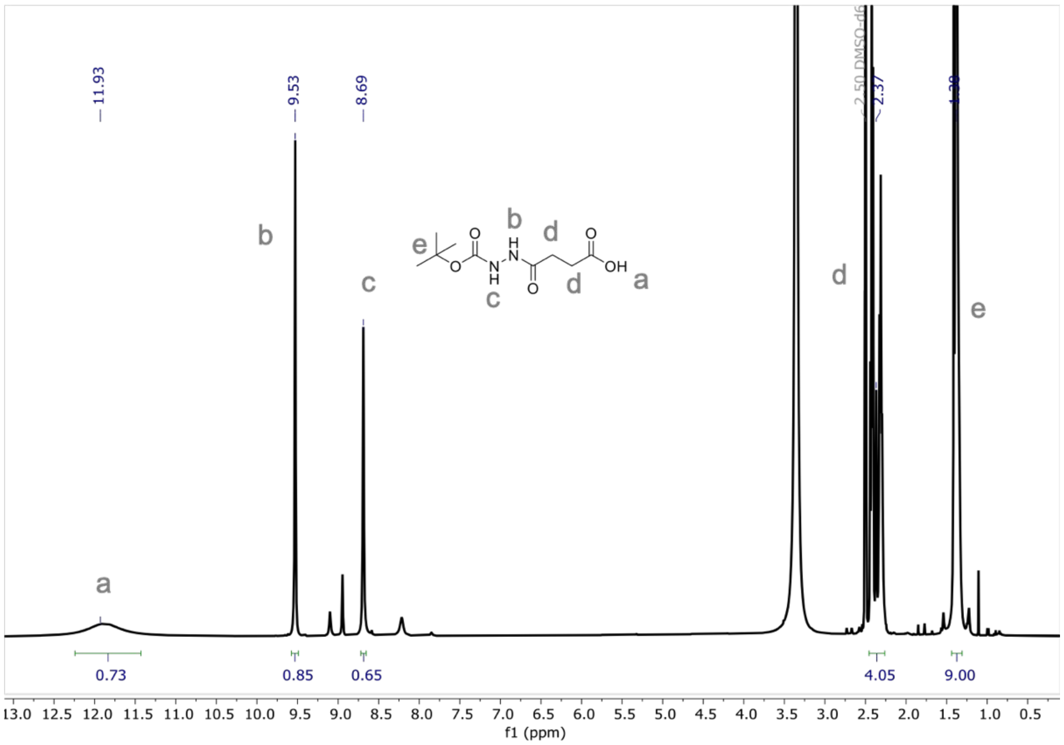

Figure S8. ^{1}H NMR of *N*-*tert*-butyloxycarbonyl-succinic acid monohydrazide (**7**) in DMSO-d_6.

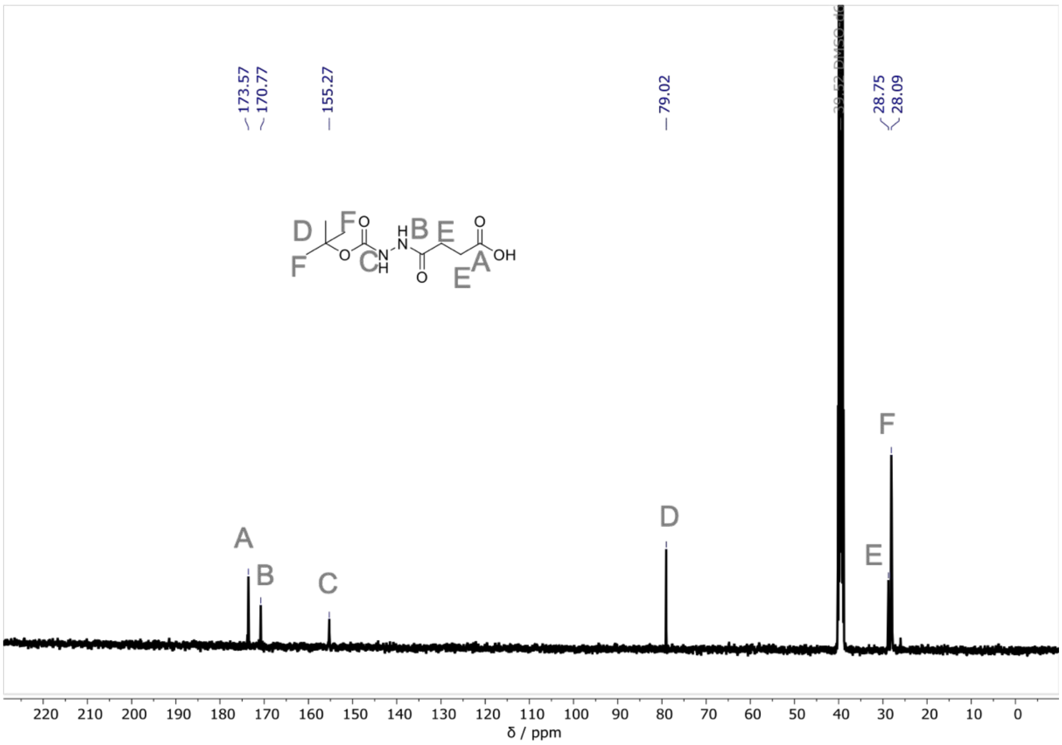

Figure S9. ^{13}C NMR of *N*-*tert*-butyloxycarbonyl-succinic acid monohydrazide (**7**) in DMSO-d_6.

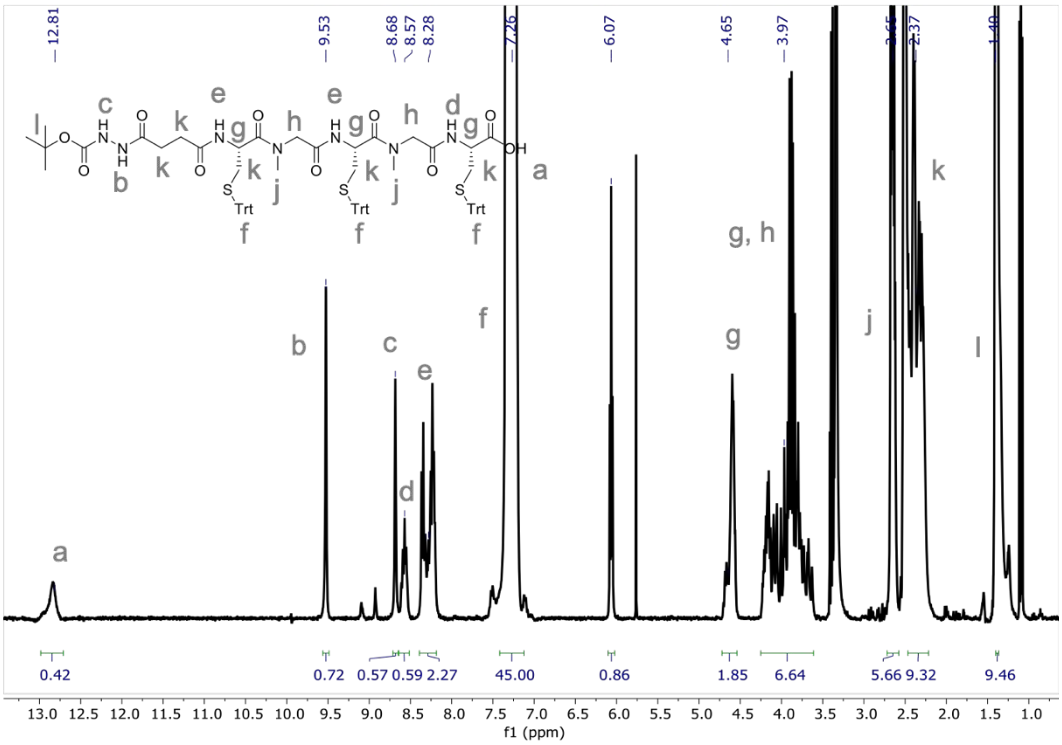

Figure S10. ^{1}H NMR of Boc-hydrazine-Cys(Trt)-Sar-Cys(Trt)-Sar-Cys(Trt)-OH (**8**) in DMSO-d_6.

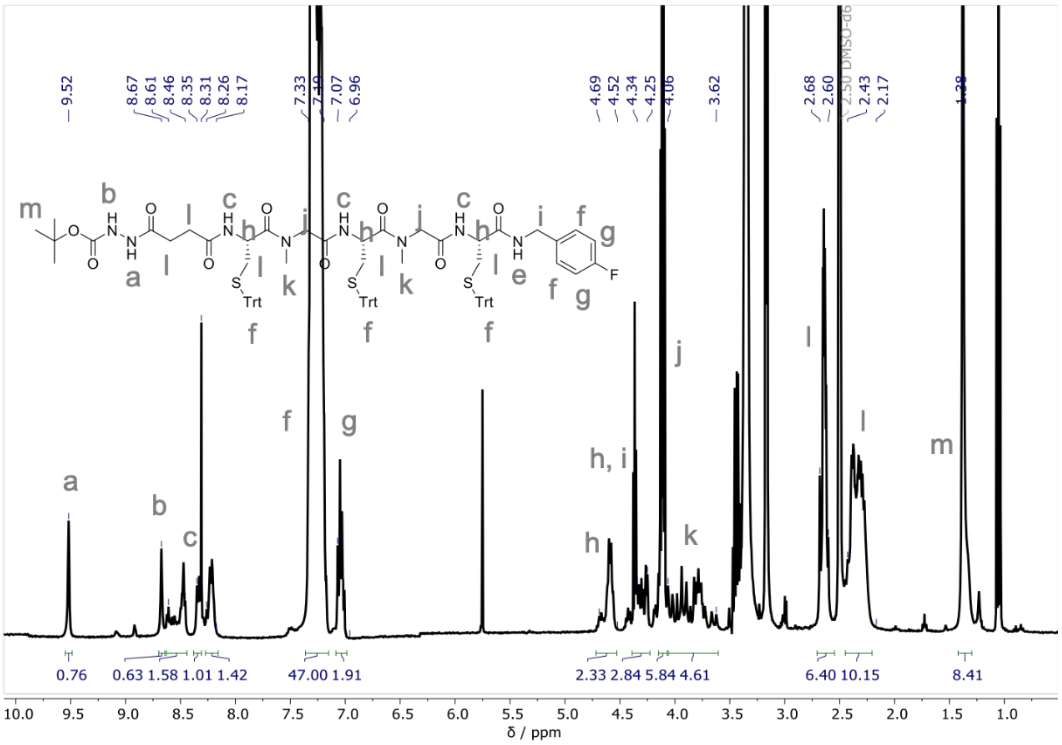

Figure S11. ^{1}H NMR of Boc-hydrazine-Cys(Trt)-Sar-Cys(Trt)-Sar-Cys(Trt)-(4-fluorobenzylamine) (**9**) in DMSO-d_6.

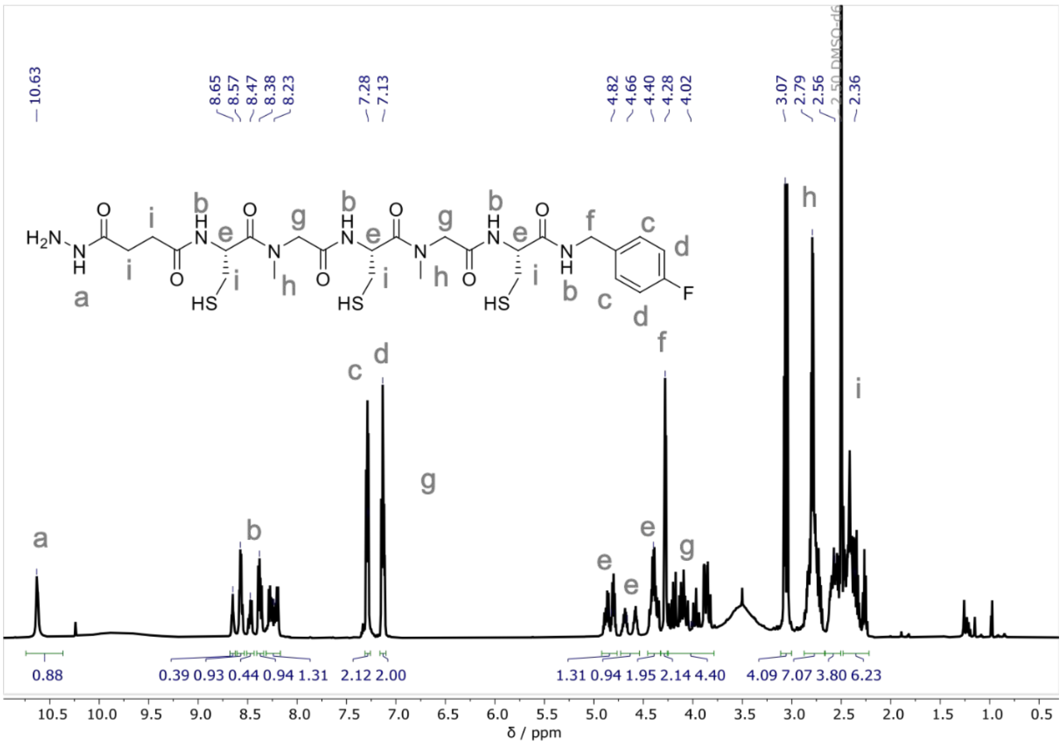

Figure S12. ^{1}H NMR of hydrazine-Cys-Sar-Cys-Sar-Cys-(4-fluorobenzylamine) (**10**) in DMSO-d_6.

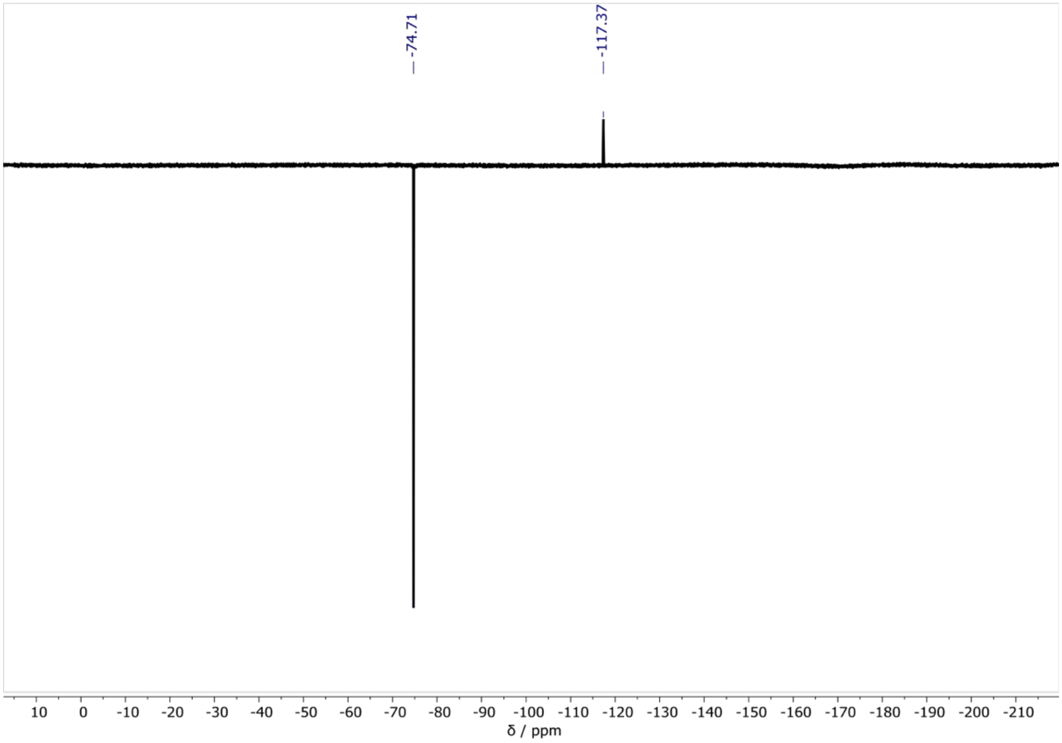

Figure S13. ^{19}F NMR of hydrazine-Cys-Sar-Cys-Sar-Cys-4-fluorobenzylamine (**10**) in DMSO-d_6.

Table S1. Analytical results of the polypept(o)ide characterization.

Polymer	X_n pSar[a]	X_n pCys(SO_2Et)[b]	M_n [c]	$Đ$ [c]
**P1**	200	-	40.9 kg mol^{-1}	1.17
**P2**	200	17	41.1 kg mol^{-1}	1.19
**P3**	200	30	44.9 kg mol^{-1}	2.52

[a] HFIP-GPC relative to pSar standards. [b] determined by ^{1}H-NMR. [c] HFIP-GPC relative to PMMA standards; secondary structure formation by p(L)Cys(SO_2Et) is not suppressed accounting for broad PDIs.
